# A systematic review of studies utilizing hair glucocorticoids as a measure of stress suggests the marker is more appropriate for quantifying short-term stressors

**DOI:** 10.1038/s41598-019-48517-2

**Published:** 2019-08-19

**Authors:** Otto Kalliokoski, Finn K. Jellestad, Robert Murison

**Affiliations:** 1 Department of Experimental Medicine, University of Copenhagen, Copenhagen, Denmark; 2 Department of Biological and Medical Psychology, University of Bergen, Bergen, Norway

**Keywords:** Neurophysiology, Diagnostic markers

## Abstract

Quantitating glucocorticoids (GCs) in hairs is a popular method for assessing chronic stress in studies of humans and animals alike. The cause-and-effect relationship between stress and elevated GC levels in hairs, sampled weeks later, is however hard to prove. This systematic review evaluated the evidence supporting hair glucocorticoids (hGCs) as a biomarker of stress. Only a relatively small number of controlled studies employing hGC analyses have been published, and the quality of the evidence is compromised by unchecked sources of bias. Subjects exposed to stress mostly demonstrate elevated levels of hGCs, and these concentrations correlate significantly with GC concentrations in serum, saliva and feces. This supports hGCs as a biomarker of stress, but the dataset provided no evidence that hGCs are a marker of stress outside of the immediate past. Only in cases where the stressor persisted at the time of hair sampling could a clear link between stress and hGCs be established.

## Introduction

Measuring glucocorticoids (GCs) deposited in hair is an increasingly popular method for biomarker-based stress assessment. Hair is sampled easily and painlessly, it is often an abundant source of material, and it has been argued to have superior qualities over other methods for analyzing GCs when it comes to gauging chronic stress^1–3^. If GCs are sequestered from the blood stream and locked into place in the growing hair at the level of the hair follicle, a single strand of hair contains within it a historical record of the HPA axis activity of its owner spanning months into the past. This idea is taken to the next level when researchers segment hairs and analyze different sections, ostensibly corresponding to different periods in the past, to make inferences regarding the perceived stress levels over time of their subjects, whether human patients^4^, captive animals^5^, or long-dead mummies^6^.

But are hair glucocorticoids (hGCs) a robust marker of stress? Local production of GCs in the hair follicle has been confirmed^7^, with the local (follicular) HPA axis appearing to respond to local stressors independently of the rest of the organism^8^. The rate of incorporation of GCs into hairs^9^ is unclear and the mechanisms by which this takes place are unknown^10,11^. Taken together, it is unclear to which degree hGCs are reflective of the (central) stress response of an individual. It has even been suggested that an individual’s hGC levels may follow a circadian rhythm, changing with the time of day^12^. Moreover, with some evidence that hGC concentrations change much faster than can be explained by mechanisms concerning only incorporation of GCs in the hair follicle^13,14^, can sections of a hair really be related to a specific period in the past?

Hair analysis for quantitating GC levels builds on a long-standing forensic practice of looking for deposition of toxins and illicit substances in hairs and fingernails^15^. Methods for detecting residual anabolic steroid hormones in hair gained momentum in the wake of a high-profile doping scandal in competitive cycling in 1998^16,17^, and were subsequently further extended for quantitating also endogenously produced steroids^18^. A study in 2002 on rock hyraxes^2^ is, we believe, the first study to infer preceding stress from the concentrations of hGCs. In 2007 (the study seeming to have been initiated years earlier^19^), the first study linking hGCs to stress was published for humans^20^, citing the method’s successful application in animals (specifically, Davenport *et al*.^1^). Notably, with a leg-up with respect to method development from forensics, none of these early papers needed to address methodological issues with respect to when and how GCs were sequestered into hairs. There are, however, crucial differences between the forensic application of hair analysis and hair analysis of GCs for estimating preceding stress. With illicit (exogenous) substances, concentrations are less important – there are no legal circulating levels of cocaine, athletes should not present with traces of nandrolone in their system, and if your hairs contain traces of thallium you have angered a regime and you are secretly being poisoned – quantitation is mostly only relevant with regards to detection limits. Endogenous compounds are more problematic in a forensic context as relative levels become important (testosterone doping in athletes being a notorious forensic conundrum^21^). Moreover, relating the position in a strand of hair of an exogenous substance of interest to a time of exposure in the past is highly approximate, even if the substance’s interactions with the hair matrix are well-characterized^22,23^. Individual differences in hair uptake (capacity) have also been noted, suggesting that comparisons of measured quantities between subjects may be inappropriate^24,25^. For hGC analyses, by contrast, concentrations, timing of deposition, and inter-individual comparisons are key features. Here, there are no clear precedents within the vast body of literature on forensic hair analysis for hGC analyses to lean on.

Despite the many unknowns surrounding the use of hGCs as a measure of chronic stress, the biomarker is presently used to gauge mental illness^26^, the wellbeing of human trauma victims^27^ (and long-term consequences of the trauma^28,29^), post-traumatic stress disorder (PTSD) sufferers^4,30,31^, and children^20,32^; to assess animal welfare in wildlife^33^, captive animals^34,35^ and laboratory animals^36^. The mismatch between the uncertainties of the method and the confidence with which it is applied is concerning.

The present systematic review strove to collect and evaluate the empirical evidence supporting the use of hGC analysis as a method for assessing physiological and psychological stress. With the method being used both for studying animals and humans, and the two bodies of literature feeding off one another, limiting the study to either humans or non-human animals would not have painted a complete picture. Unlike previous reviews/meta-analyses^3,26,37,38^, we thus set out to collate data from all vertebrate species; to include studies carried out in human and non-human animals alike. Specifically, we investigated whether hGC levels had been found to correlate well with concentrations of GCs in other biological matrices, proven to be reflective of HPA axis activity in the recent past. We collated studies that compared hGC levels in stressed individuals to those of unstressed controls to determine whether an up-regulated HPA axis would produce a predictable increase in hGCs. Through meta-analytic investigation of these two sets of studies, we attempted to answer whether hGC concentrations appear to be reflective of the central HPA axis activity. Specifically, the focus in this review is to assess the evidence supporting the claim that stressful stimuli result in measurable increases in hGC levels. Moreover, by subdividing controlled studies by type and temporality of the stressor, we attempted to determine whether hGC levels are better suited for describing certain types of stress than others.

## Material and Methods

The methods listed below were pre-specified in a study protocol accessible online since Jan 13, 2016 (Supplemental materials C).

A broad – inclusive – search strategy was employed in an attempt to find all relevant publications that could provide unambiguous evidence of hGCs being related to the HPA axis-activating stress response of an individual. Studies where hGC levels were used as a measure of stress or where hGC levels were correlated to GC levels in other biological matrices (blood, saliva, urine and feces) were retrieved through multiple databases (MEDLINE, Web of Science, EMBASE, Zoological Record, and PsycINFO). The publication searches were conducted in January 2016. Whereas we have contextualized our findings with more recent examples, no studies published past this date were included in the analyses. Quality assessments were made according to an adapted (nine-item) checklist and basic study information was extracted along with hGC results. Synthesizing data from multiple sources, summary estimates were created separately for correlations with different biological matrices. Similarly, experimental designs deemed fundamentally incompatible were separated out and individual summary estimates were created. Specifically, studies were classified as studying “induced (acute) stress”, “chronic stress”, “observed stress” (where the stressor was inferred by an observer), “self-assessed stress”, “past stress” (where a subject was exposed to a stressful period, which had subsequently ended prior to hair collection) and post-traumatic stress disorder (“PTSD”; which we chose to include because its link with hGCs was receiving great attention at the time this review was scoped). Random effects models were used throughout and differences in experimental subjects and controls were expressed as standardized mean differences. For detailed descriptions of the methods, refer to Supplemental materials A.

## Results

**Figure 1:**
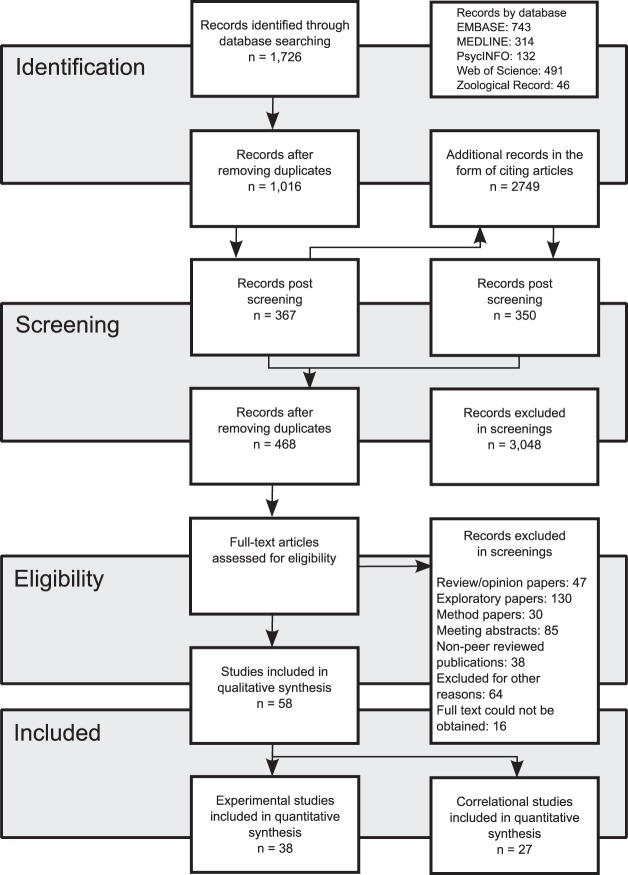
Flow chart outlining the systematic search strategy, the subsequent screening, and inclusion/exclusion of database entries. The diagram has been adapted from the PRISMA Flow Diagram^87^.

A total of 3,518 unique entries were found using the search strategy, of which 468 entries were retained for full text analysis (Fig. 1). A majority of these studies were subsequently excluded due to not meeting the pre-stated inclusion criteria: 28% were excluded due to their exploratory study design – often characterized by the lack of a control group and a clear *a priori* hypothesis; 16% presented no data from a controlled study – these were mostly method papers, reviews, opinion papers, and other narrative journal entries; 26% were not peer-reviewed publications – these were mostly meeting abstracts and theses. Other incompatible study designs, and entries where the full text could not be obtained, made up 17% of the entries retained for full text screenings.

For the entries retained for full text screening – where all texts were verified to concern the use of hGCs – an exponential growth in method adoption is obvious: 2015 saw more publications on hGCs than had been published between 2003 and 2011 in total. Presently, a new publication (counting also non-peer reviewed entries) on hGCs is available online every three days (or less).

### Study quality of experimental studies

Of the 59 peer-reviewed publications included in the present systematic review, 38 papers reported on 42 studies with a stress group/control group design that could be assessed for study quality.

**Figure 2:**
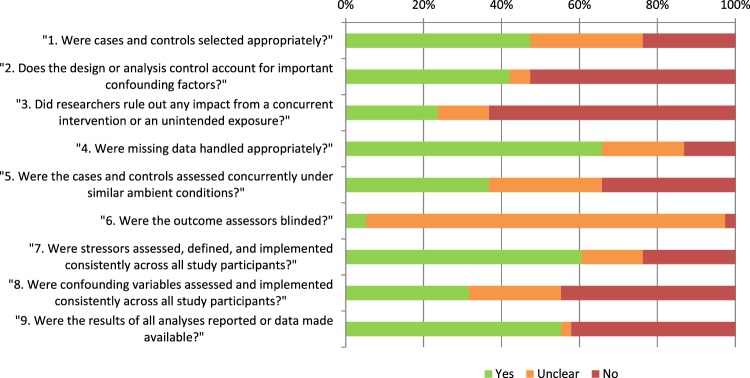
Results from the risk-of-bias checklist assessment of the experimental study designs.

A salient trend was found when assessing the risk of bias: A majority of the 38 papers did not account for the possibility that a stressor other than the one that was purportedly studied could have influenced the results. This is evident in Fig. 2 focusing on checklist items 2, 3 and 8: The influence of concurrent interventions or unintended exposures could only be ruled out in 9 (24%) of the studies (item 3), the influence of confounding factors could only be ruled out in 16 (42%) of the studies (item 2), and only 12 (32%) of the studies featured a study design that ensured that the subjects were equally exposed to any confounding factors (item 8). In only three studies (8%) could all three sources of bias be ruled out entirely. Similar ambient conditions for stress and control groups could also only be guaranteed in 15 (39%) of the studies (item 5). Remarkably, only 3 (8%) of the studies reported on blinding of the outcome assessors (item 6), even though this is an explicit recommendation of most present-day best-practice frameworks (e.g. the ARRIVE guidelines^39^). In no one study were all of the sources of bias addressed, and in a few none were (for a by-entry summary of the risk-of-bias analyses, refer to Supplemental materials B, appendix 1).

### Study characteristics and data extraction

The studies retained for analysis presented a diverse set, with no two study designs quite alike (Tables 1 and 2). Of the studies retained for analysis, roughly half (48%) were human studies. Both sexes have been studied in roughly equal numbers (52% female subjects across all studies), but only rarely were equal sex ratios employed in any one study; study objectives and opportunistic sampling of e.g. wildlife populations tending to bias the sex ratio in favor of one or the other. We made initial attempts at exploring sex differences – similar to a previous meta-analysis^38^ – however the data were insufficient to draw any conclusions. Similarly, when extracting data we had harbored hopes of being able to compare the effects of differing sampling and analysis protocols that have been discussed previously^40^. However, the laboratory methods employed were fairly similar and study designs fairly dissimilar, the combination lending itself poorly to stringent analyses. Human studies were consistent in sampling the posterior vertex of the head, whereas the non-human studies appeared to sample regions by convenience or just by random (e.g. studies in dogs have sampled backs, shoulders, chests, and legs, depending on research group and study). Although often discussed as a potential issue^41,42^ no one study admitted to including hair follicles in their hair samples and all but six papers^5,34,43–46^ explicitly described methods designed to ensure samples being free of follicles. It has been theorized that the color of a hair can influence the GC content^47^, however the results are inconsistent^10^ and hair color is rarely reported. We consequently did not attempt to extract information on this. Only human and other primate studies employed the “stress calendar” idea, sub-sectioning hairs to infer circulating GC levels at multiple time points in the past from the same sample. Of all the studies retained for analysis, a clear majority (48 studies, 83%) employed a washing step, intended to remove contaminants from the outside of the hairs, and all but two studies minced/pulverized the hairs prior to analysis. For quantification of GCs, antibody-based methods were most frequently employed (48 studies, 83%), however numerous different protocols/antibodies have been utilized.

**Table 1:** Study characteristics for studies with extracted correlations. In total, 29 comparisons were extracted from 27 peer-reviewed publications, collecting data from 1,093 subjects across nine species. These studies were used for producing summary estimates of correlation coefficients between hGC concentrations and GC concentrations in other biological matrices.

Correlational studies	Subjects	Correlation with GCs in	Males/females/unknown	Sampling site	* = only section nearest to the scalp was analyzed				Analysis method
Study					Sub-segmentation?	Follicle?	Wash?	Processing?	
Accorsi, 2008	Cats	Feces	8/19/0	Back (ischiatic region)	No	No	No	Cut	RIA
Accorsi, 2008	Dogs	Feces	21/8/0	Back (ischiatic region)	No	No	No	Cut	RIA
Bennett, 2010	Dogs	Saliva	0/0/48	Back (ischiatic region)	No	No	Yes	Milled	ELISA
Bryan, 2013a	Dogs	Feces and saliva	5/2/0	Shoulders	No	No	Yes	Milled	ELISA
Chan, 2014	Humans	Serum, urine and saliva	26/31/0	Head	No	No	No	Cut	ELISA
Chen, 2014	Humans	Urine	29/0/0	Head (posterior vertex)	No	No	Yes	Milled	HPLC-MS/MS
Corradini, 2013	Dogs	Serum	49/41/0	Chest (xiphoid region)	No	No	No	Cut	RIA
D’Anna-Hernandez, 2011	Humans	Saliva	0/21/0	Head (posterior vertex)	Yes	No	Yes	Milled	ELISA
Davenport, 2006	Rhesus monkeys	Saliva	20/0/0	Neck (posterior vertex)	Yes	No	Yes	Milled	ELISA
Kuehl, 2015	Humans	Saliva	31/54/0	Head	Unclear	No	Yes	Unclear	CLIA
Manenschijn, 2012	Humans	Saliva	0/0/90	Head (posterior vertex)	No	No	No	Cut	ELISA
Mastromonaco, 2014	Chipmunks	Feces	0/0/62	Leg	No	No	Yes	Cut	ELISA
Moya, 2013	Cattle	Saliva and feces	12/0/0	Head, neck, shoulder, hip, tail	No	Unclear	Yes	Milled	ELISA
Moya, 2015	Cattle	Saliva	80/0/0	Tail	No	Unclear	Yes	Milled	ELISA
Ouschan, 2013	Dogs	Blood	4/8/0	Leg (inside)	No	No	Yes	Cut	ELISA
Pulopulos, 2014	Humans	Saliva	12/38/0	Head (posterior vertex)	No	No	Yes	Milled	LC-MS/MS
Sauvé, 2007	Humans	Saliva, urine and serum	19/20/0	Head (posterior vertex)	No	No	No	Cut	ELISA
Schalinski, 2015	Humans	Saliva	0/28/0	Head (posterior vertex)	Yes	No	Yes	Unclear	CLIA
Steudte, 2011	Humans	Saliva	0/27/0	Head (posterior vertex)	Yes	No	Yes	Milled	CLIA
Steudte, 2013	Humans	Saliva	6/72/0	Head (posterior vertex)	Yes	No	Yes	Milled	LC-MS/MS
Sumra, 2015	Humans	Urine	0/31/0	Head	No*	No	Yes	Ground	ELISA
Tallo-Parra, 2015	Cattle	Feces	0/17/0	Head	No	No	Yes	Cut	ELISA
van Holland, 2012	Humans	Saliva	0/0/27	Head	No	No	Yes	Milled	CLIA
Vanaelst, 2012	Humans	Saliva and serum	0/32/0	Head (posterior vertex)	No*	No	No	Cut	LC-MS/MS
Wippert, 2014	Humans	Urine	3/10/0	Head (posterior vertex)	No	No	Yes	Unclear	LC-MS/MS
Xie, 2011	Humans	Saliva	32/0/0	Head (posterior vertex)	No*	No	Yes	Milled	LC-MS/MS
Yamanashi, 2013	Chimpanzees	Feces	9/0/0	Arm	No	No	Yes	Milled	ELISA
Yu, 2015	Mice	Serum	19/0/0	Back	No	No	Yes	Cut	LC-MS/MS
Yu, 2015	Rats	Blood	22/0/0	Back	No	No	Yes	Cut	LC-MS/MS

**Table 2:** Study characteristics for experimental studies. In total, 42 comparisons were extracted from 38 peer-reviewed publications, collecting data from 3,199 subjects across 16 species. These studies were used for creating summary estimates for the difference in hGC concentrations between stressed subjects and controls for different sub-categories of stressors.

Experimental studies	Subjects	Stressor	Classification	Males/females /unknown	Sampling site	* = only section nearest to the scalp was analyzed				Analysis method
Study						Sub-segmentation?	Follicle?	Wash?	Processing?	
Ashley, 2011	Caribou	ACTH challenge	Past stress	6/6/0	Neck, shoulder, rump	No	Unclear	Yes	Milled	ELISA
Ashley, 2011	Reindeer	ACTH challenge	Past stress	6/6/0	Neck, shoulder, rump	No	Unclear	Yes	Milled	ELISA
Boesch, 2015	Humans	Military training	Chronic stress	105/0/0	Head (posterior vertex)	No*	No	Yes	Cut	ELISA
Bryan, 2013b	Bears	Anthropogenic disturbance	Chronic stress	87/25/0	Unknown	No	Unclear	Yes	Milled	ELISA
Bryan, 2015	Wolves	Anthropogenic disturbance	Chronic stress	76/72/30	Unknown	No	No	Yes	Milled	ELISA
Carlitz, 2014	Orangutans	Various, undefined	Observed stress	31/27/0	Mixed/unclear	Yes	Unclear	Yes	Cut	CLIA
Cattet, 2014	Bears	Capture stress	Induced (acute) stress	240/246/0	Mixed/unclear	No	No	Yes	Milled	ELISA
Chu, 2014	Cynomolgus monkeys	Post-partum depression	Observed stress	0/10/0	Back	No	No	Yes	Milled	RIA
Corradini, 2013	Dogs	Hypercortisolism	Chronic stress	49/41/0	Chest (xiphoid region)	No	No	No	Cut	RIA
Davenport, 2006	Rhesus monkeys	Relocation stress	Induced (acute) stress	20/0/0	Neck (posterior vertex)	Yes	No	Yes	Milled	ELISA
Dettenborn, 2010	Humans	Long-term unemployment	Chronic stress	13/46/0	Head (posterior vertex)	Yes	No	Yes	Milled	CLIA
Dettmer, 2014	Rhesus monkeys	Crowding stress	Chronic stress	0/0/152	Neck (posterior vertex)	No	No	Yes	Milled	ELISA
Fairbanks, 2011	Vervet monkeys	High stress environment	Induced (acute) stress	0/226/0	Back	No	No	Yes	Milled	ELISA
Fourie, 2015	Vervet monkeys	Anthropogenic disturbance	Chronic stress	29/43/0	Back	No	No	Yes	Cut	ELISA
Gao, 2014	Humans	Traumatic event	Self-assessed stress	75/17/0	Head (posterior vertex)	No	No	Yes	Milled	LC-MS/MS
González-de-la-Vara 2011	Cattle	ACTH challenge	Induced (acute) stress	0/15/0	Unclear	No	No	Yes	Cut	RIA
Heinze, 2016	Humans	Mental health problems	Self-assessed stress	6/52/0	Head (posterior vertex)	Yes	No	Yes	None	CLIA
Henley, 2013	Humans	Socioeconomic stress	Chronic stress	25/15/32	Head (posterior vertex)	No	No	Yes	Cut	ELISA
Jarcho, 2016	Mice	Social instability stress	Induced (acute) stress	0/24/0	Back (lower back)	No	No	Yes	Unclear	ELISA
Kapoor, 2016	Rhesus monkeys	Acoustic startle stress	Past stress	0/35/0	Back (upper back)	No	No	Yes	Milled	LC-MS/MS
Karlén, 2011	Humans	“Serious life-event”	Self-assessed stress	24/71/0	Head (posterior vertex)	No*	No	No	Milled	RIA
Klumbies, 2014	Humans	Social phobia	Observed stress	0/0/53	Head (posterior vertex)	No*	No	Yes	Unclear	CLIA
Luo, 2012	Humans	Traumatic event	Observed stress	0/84/0	Head (posterior vertex)	Yes	No	Yes	Milled	CLIA
Luo, 2012	Humans	Post-traumatic stress disorder	PTSD		Head (posterior vertex)	Yes	No	Yes	Milled	CLIA
Manenschijn, 2011	Humans	Shift work	Chronic stress	122/0/0	Head (posterior vertex)	No	No	No	Unclear	ELISA
Mastromonaco, 2014	Chipmunks	ACTH challenge	Induced (acute) stress	0/0/12	Leg	No	No	Yes	Cut	ELISA
Moya, 2015	Cattle	Digestive problems	Induced (acute) stress	80/0/0	Tail	No	Unclear	Yes	Milled	ELISA
Nejad, 2014	Sheep	Water restriction	Induced (acute) stress	0/9/0	Neck (posterior vertex)	No	No	Yes	Cut	ELISA
Oullette, 2015	Humans	Psychosocial stress	Self-assessed stress	0/60/0	Head (posterior vertex)	No*	No	Yes	Cut	ELISA
Qin, 2015	Rhesus monkeys	Induced SADS	Chronic stress	0/8/0	Back	No	No	Yes	Milled	RIA
Schalinski, 2015	Humans	“Stress-related disorders”	PTSD	0/51/0	Head (posterior vertex)	Yes	No	Yes	Unclear	CLIA
Scorrano, 2015	Rats	Misc. stress protocols	Induced (acute) stress	58/0/0	Back (lower back)	No	No	Yes	Ground	RIA
Skoluda, 2012	Humans	Intensive training	Chronic stress	144/151/0	Head (posterior vertex)	Yes	No	Yes	Milled	CLIA
Stalder, 2014	Humans	Caring for relative with dementia	Chronic stress	4/36/0	Head (posterior vertex)	No*	No	Yes	Milled	CLIA
Steudte, 2013	Humans	Traumatic event	Self-assessed stress	6/72/0	Head (posterior vertex)	Yes	No	Yes	Milled	LC-MS/MS
Steudte, 2013	Humans	Post-traumatic stress disorder	PTSD		Head (posterior vertex)	Yes	No	Yes	Milled	LC-MS/MS
Steudte-Schmiedgen, 2015	Humans	Military deployment	PTSD	90/0/0	Head (posterior vertex)	No*	No	Yes	None	LC-MS/MS
Terwissen, 2013	Lynxes	ACTH challenge	Induced (acute) stress	1/2/0	Unclear	No	Unclear	Yes	Cut	ELISA
van Uum, 2008	Humans	Chronic pain	Chronic stress	25/29/0	Head (posterior vertex)	No*	No	No	Cut	ELISA
Yamada, 2007	Humans	Post-birth complications in neonates	Chronic stress	0/0/78	Head	No	No	No	Cut	ELISA
Yu, 2015	Mice	Aggression/social instability	Induced (acute) stress	19/0/0	Back	No	No	Yes	Cut	LC-MS/MS
Yu, 2015	Rats	Surgery/post-surgical pain	Induced (acute) stress	22/0/0	Back	No	No	Yes	Cut	LC-MS/MS

When extracting data, two studies – Manenschijn *et al*.^48^ and Luo *et al*.^4^ – were singled out as having a reported precision more than tenfold higher than the other 38 studies (including studies utilizing the very same methodology in comparable subjects). We believe that this is simply due to incorrect reporting of the measure of dispersion. Unable to reach the authors for a comment – despite multiple attempts – we have tentatively included the data from these studies, assuming that the graphically presented measures of dispersion were in fact SEMs, rather than – as listed – 95% CIs. Three studies – two experimental studies of chronic stressors^33,49^, and one study reporting a non-significant correlation between hGC and GC in saliva^50^ – were excluded from further analysis as critical information could not be obtained from the corresponding authors (none of the studies listed the number of samples/subjects used in their analyses). We do not expect these exclusions to have significantly altered our summary estimates, however, as these studies were of moderate size and all fell into well-populated subgroups.

### Correlations with GC in other matrices

**Figure 3:**
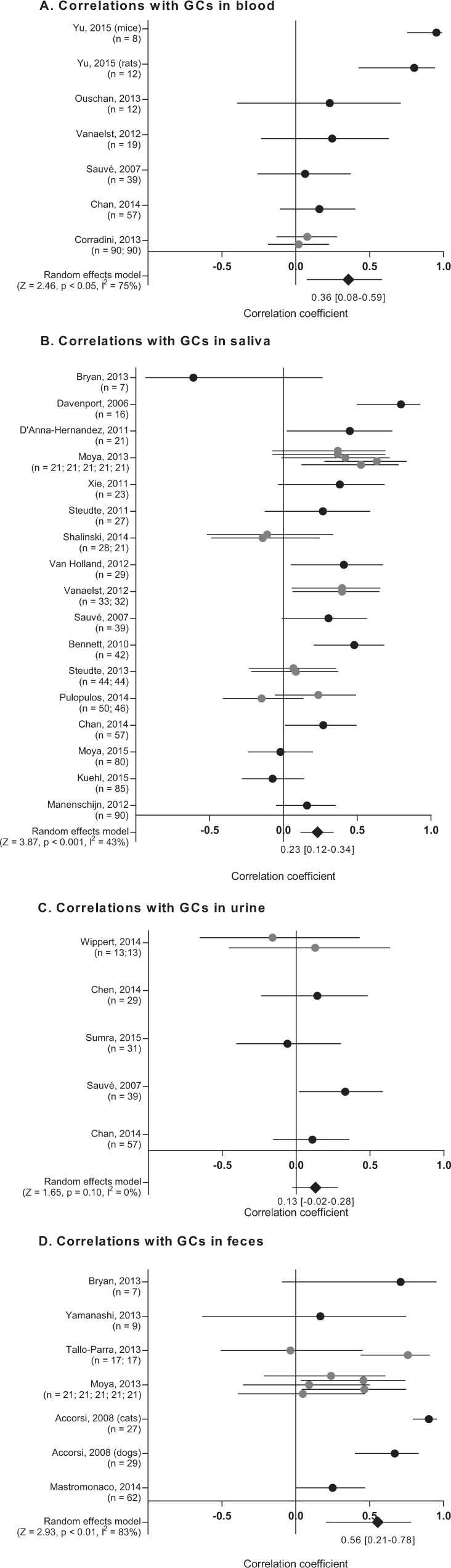
Synthesis of correlation coefficients. Forest plots are presented for correlation coefficients between hGCs and GC in (**A**) blood, (**B**) saliva, (**C**) urine, and (**D**) feces. Where multiple coefficients were reported in the same study (grey markers) these were weighted accordingly, to avoid biasing the random effects model. The summary estimate correlation coefficients are reported with 95% CI.

Meta-analyses of correlation coefficients revealed a great deal of heterogeneity between studies, as could be expected from the diverse set of studies analyzed (Fig. 3). Significant synthesized meta-correlations could be found between hGCs and GCs in blood, saliva and feces. A significant correlation could not be found between hGCs and GCs in urine. However this analysis featured only five studies (collecting 169 subjects), all with fairly high intra-study variance of data. Leave-one-out analysis furthermore revealed that the statistically significant correlation found between GCs in blood and hGCs could not be substantiated if data from the study by Yu *et al*.^51^ were removed. Moreover, removing the data from the study by Accorsi *et al*.^52^ would more than halve the synthesized correlation coefficient between GCs in feces and hGC (putting it in range with the other correlations at r = 0.22), suggesting that the strength of the correlation may be somewhat overestimated. Additionally, removing a single study for each correlation summary would reduce heterogeneity markedly, bringing down the largest I^2^ value to 38%, suggesting a small number of studies were responsible for a majority of the heterogeneity (the leave-one-out analyses are presented in their entirety in Supplemental Materials B, Appendix 2). A factor adding to the heterogeneity may also be that some studies averaged multiple samples over time – e.g. the study by D’Anna-Hernandez *et al*.^53^ where hair and saliva samples were averaged across four sampling points throughout human pregnancy, or Sauvé *et al*.^54^ comparing single hair samples to urine samples averaged over a 24-hour period. With a relatively small dataset, we did not see fit to analyze these as separate subgroups, increasing our “researcher degrees of freedom”^55,56^, and potentially flagging spurious correlations (this would, further, only have been possible for the correlation with GC in saliva). Moreover, the studies that correlated both point estimates and averages^31,57–59^ did not demonstrate a consistent difference between the two approaches.

### hGCs as a measure of stress

**Figure 4:**
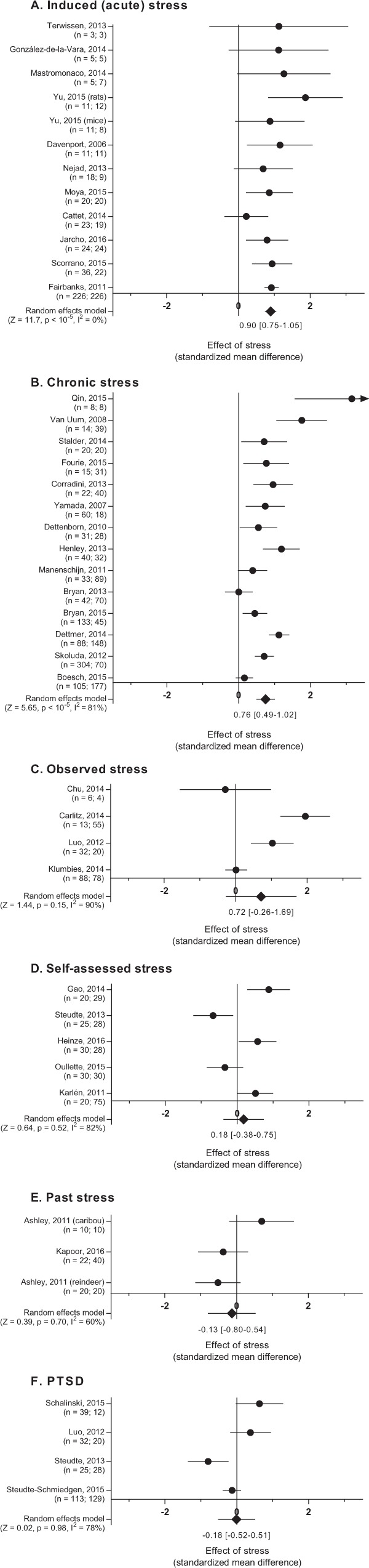
Forest plots summarizing results from induced (acute stress) studies (**A**), chronic stress studies (**B**), studies where stress was inferred through observation (**C**) or assessed by the subject (**D**), studies where the stressor had passed (**E**), and studies featuring subjects with PTSD (**F**). The number of subjects in the studies are listed with the control group last. Summary estimates (Hedges’ g) were constructed using the DerSimonian-Laird approach and are reported with 95% CI.

Summarizing the evidence from the experimental studies using random effects models produced varied results (Fig. 4), reaffirming our decision to carry out subgroup analyses. Induced (acute) stress models produced a clear elevation in GC concentrations measured in hairs (Fig. 4A) with low inter-study heterogeneity (I^2^ could not be estimated). Chronic stressors also produced a significant elevation in deposited GC compared to control groups (Fig. 4B). The results from the chronic stress studies were however highly heterogeneous with a majority of the variance of the summary estimate stemming from between-study variation, as opposed to within-study variation (I^2^ = 80%), suggesting that not all of the studies were comparable with respect to the stress response and its effect on hGC levels. Simply put, it is unlikely that these studies all describe a similar HPA axis activation in response to the studied stressor; it is, for instance, likely that some scenarios simply did not induce a stress response. Observed stress (Fig. 4C) and self-assessed stress (Fig. 4D) produced unclear results. Finally, stressors that had subsided at the time of sampling (“past stress”) did not produce a measurable elevation in hGCs (Fig. 4E). Studies concerning hGCs measured in PTSD sufferers similarly generated unclear results (Fig. 4F), with a combination of studies showing both elevations and decreases in hGC output relative to a control group.

## Discussion

### Study landscape as evidenced by systematic searches

With a couple of new papers appearing every week that concern or utilize hGC analysis, it is fair to say that it has become a widespread method for assessing stress. But hair-growth is a slow process, and popular speculation^60,61^ suggests that GCs are sequestered by hairs over several weeks – if not months. Consequently, controlled studies are for logistic reasons hard to design and execute. Perhaps this is why our search strategy turned up more narrative reviews, opinion papers, and book chapters lauding the method than it did actual controlled studies providing empirical evidence that the method is a sound one. Moreover, the typical (i.e. the most numerous) study employing hGC analyses, published prior to February 2016, was an exploratory one. Characteristically, a single cohort of subjects had hair samples collected along with a number of other environmental, physiological, psychological, and/or demographic data. Correlations were then constructed to scrutinize which parameters were linked to elevated hGC concentrations. The topics of these studies are varied, from investigations of environmental effects on squirrel gliders^62^ or social effects on German shepherds^63^ to probing cultural^64^, environmental^65^, nutritional^32^ or genetic^66^ influences on psychological stress in people of differing ages. The implicit prior assumption for studies of this kind is that hGCs are linked to central HPA axis functioning and are thus a measure of (chronic) stress. This puts even more of an onus on the (relatively small number of) controlled studies to validate and affirm the use of hGC concentrations as a measure of stress.

### Is there support for hGCs as a biomarker of stress?

The present investigation supports the use of hGCs as a measure of central HPA axis functioning and, consequently, as a stress-sensitive biomarker. The compounded data however calls into question the temporality of the marker, suggesting it is a better marker for ongoing than of past stress.

In studies where subjects were exposed to a controlled stressor, a predictable elevation was found in most cases. Whether repeated ACTH challenges^67^ or a more elaborate protocol combining multiple stressors were employed^68^, a consistent increase was found across species when comparing challenged subjects to unstressed controls. The effect of acute stress on hGC levels seems, furthermore, to be rapid. Whereas most studies sampled hairs at least two weeks after having applied the stressor, the study by Cattet *et al*.^14^ is remarkable in that they report elevated hGCs within hours of stressor onset. In a similar vein, most stress protocols were applied continuously for weeks before hGC concentrations were evaluated, but González-de-la-Vara *et al*.^67^ found that they could detect an elevation in hGCs two weeks after a pair of sustained-release ACTH injections. Both studies point to hGC concentrations being reflective, primarily, of events in the recent past, as opposed to historical stressors. This is also consistent with hGCs correlating with GCs in other matrices.

Although both inter- and intra-study variances were high for the collated data, it is clear that hGC concentrations correlate significantly with GC concentrations in other matrices. The synthesized correlation coefficients are weak to moderate – ranging from 0.13 to 0.56 – but this is in range with the correlations between established matrices obtained in these very same studies^54,59,69,70^. Due to large fluctuations stemming from the pulsatile nature of GC release^71^, coupled with the different temporality of the matrices – serum and saliva concentrations of GCs change in a matter of minutes in response to a stressor, urinary and fecal GCs change over a period of hours^72^ – these correlations will inevitably be moderate at the most. The correlation between hGCs and GCs in feces is the strongest of the four, which is to be expected as fecal samples integrate circulating GC concentrations over a period of several hours. Hairs are similarly suggested to sequester GCs from circulation over a longer time window. In the face of popular claims, it is unlikely that this time window is several weeks long, however, as hGC concentrations also correlate significantly with serum and salivary concentrations of GCs.

### The effect of confounders on measured hGC levels in chronic stress studies

When compiling studies of chronic stress, a link between individuals experiencing stress and elevated levels of hGCs was found, albeit a weaker link than for acute stressors. The greater level of heterogeneity of this dataset is probably in part because some of the studies were carried out under highly uncontrolled circumstances. With long-term studies featuring subjects – whether human or non-human – in an uncontrolled environment, it is hard to ensure that the studied stressor is the sole and most influential source of stress. It may be that a lack of dietary salmon elicits a physiological stress reaction in grizzly bears, as suggested by Bryan *et al*.^43^, but it is quite impossible to tell what other factors might influence the life and allostasis of these bears. The confounding factors of this study may well have overshadowed the effect the authors were looking for. Similarly, military training is not all long marches and adrenaline-fueled combat training. With no outside verification, the soldiers undergoing basic training studied by Boesch and collaborators^73^ may not have had a more active HPA axis than e.g. an office worker with an active lifestyle in the period of sampling. This is not to criticize these experiments; rather, this is to highlight the fact that a number of studies into chronic stressors have an exploratory element to them, as the magnitude of the chronic stressor is hard to judge in relation to a host of ambient stressors. Our risk-of-bias assessment singled out unrelated confounding factors as the most common unchecked source of bias. Only 24% of studies could account for external confounding factors in the studied period, and only in 32% of studies could they be assumed to have been distributed equally between the studied subject groups. The evidence supplied by the chronic stress studies should thus be interpreted carefully.

### Differences between the human concept of stress and HPA axis activity

In our investigation, studies where periods of stress were inferred presented highly heterogeneous data. It has been shown before that when human subjects are asked to introspectively assess their own level of stress, assessments correlate poorly with their actual HPA axis functioning^38,74,75^. In the present investigation we see a similar trend for studies relying on self-reported stress. Whereas we will note that the present investigation contains only a handful of studies, no consistent trend or even weak effect can be inferred. This is not to say that the subjects were not experiencing psychological stress – the studies collect data from distressed subjects ranging from survivors of natural disasters^76^ to patients sourced from mental health services^31,77^ – but it serves as a reminder that the human concept of stress is not synonymous with the prototypical fight-or-flight response. Different states of stress will involve the HPA axis differently. This is further exemplified by the studies of PTSD, where in two studies^31,78^ a notable reduction in hGCs is found for PTSD subjects when compared to healthy controls. We specifically analyzed PTSD studies separately as it has been suggested that PTSD is accompanied by a lowering in circulating GC levels, as opposed to an elevation. Notably, PTSD subjects are identified through clinical scores suggesting the subjects were assigned to groups according to arbitrary cutoffs in a continuum of chronic stress diagnoses. This muddying of the waters, where the line between chronic stress conditions and PTSD is blurred, may, in part, explain why no clear trend is found concerning hGC profiles for either. In the future, a larger dataset that would allow for a more stringent subgrouping of chronic stress studies based on e.g. clinical scores may assist in identifying more uniform profiles.

Studies where the stressed subjects were identified through observation similarly did not paint a consistent picture. Whether the result of studying animal behavior^5,79^ or of putting human patients through structured interviews^4,80^, there seems to be a mismatch between subjectively assessed stress and hGC levels. For this category of studies we will note that it is particularly concerning that no blinding was employed, even though the findings hinge completely on the subjective assessment of an external observer. Would the marked difference between groups have been as profound in the study by Carlitz *et al*.^5^ (the average effect size was greater than that found in any induced stress study) if periods of stress had been recorded by a blinded observer? The study was made even more subjective and bias-prone by not ever defining the studied stressors, leaving it in the hands of unblinded observers to determine what to consider a stressor. With few studies and heterogeneous results, it is currently hard to determine whether studies that rely on externally assessing stress can provide empirical evidence with respect to the utility of hGC analyses.

### Temporal aspects of hGCs as a biomarker of stress

An important factor shared between the chronic stress studies that demonstrate a clear difference between stressed and control subjects is that the stressor persisted at the time of sampling. When singling out the studies where the stressor could be positively ensured to have subsided at the time of hair sampling, the pattern was found to be different. In the study by Kapoor *et al*.^81^, pregnant rhesus monkeys were exposed to a daily acoustic startle stress protocol for five weeks. Serum samples analyzed for circulating GC levels were used to verify that the protocol elicited a significant stress response throughout the period. When analyzing hGC concentrations 3–13 weeks later (depending on subject), no elevation could be found relative to a control group; not a trace to be found of a considerable elevation of circulating GC levels persisting for five weeks. Similarly, when Ashley *et al*.^34^ analyzed hairs from both reindeer and caribou two weeks after a single (non-sustained-release) ACTH challenge, no elevation could be found. Fecal GC analyses confirmed that the stressor had subsided after 24–48 hours. With only two studies in this category, we should be careful not to over-interpret; however, this is all part of a recurring pattern. In a recent meta-analysis, Stalder *et al*.^38^ reanalyzed historical data from human studies in aggregate – collecting data from 66 studies and more than 10,000 hGC samples – and found that in cases of past/absent stress, no relation with elevated hGC concentrations could be found. The idea of hairs containing a historical record of past stress is, and remains, completely unproven, empirical evidence instead pointing to hGCs being a measure of concurrent stress.

Evidence that hGC concentrations are a historical record of stress could also come in the form of studies sub-sectioning hairs, inferring circulating GC levels at multiple time-points in the past. However, the GC levels of hairs were found to be similar across all the sampled segments – individuals with elevated levels of hGCs had higher levels of hGCs in all segments when compared to controls^77,82,83^. In only two studies the authors attempted to construct a narrative based on point-to-point fluctuations in GC concentrations along the hair shafts. The findings by Luo and collaborators^4^ are however marred by a strong wash-out effect, with hGC levels successively becoming lower the further away from the scalp a segment is sourced. The most distant segment is purported to contain the lowest levels of hGCs as this segment is hypothesized to correspond to a period before a major trauma. However, this also holds true for the non-traumatized controls, undermining the hypothesis. The study by Carlitz *et al*.^5^ is similarly problematic in that the narrative seems to have been constructed *post hoc*, and only three individual profiles are shown in the paper. To our knowledge, there is little evidence to suggest that interactions between a steroid hormone and a strand of hair are strong enough to lock the molecules permanently in place. This effect is the basis by which a specific section of hair can be related to a time-point in the past. Convincing evidence that baleen from whales can trap hormones, leaving a historical record of hormonal fluctuations, has been presented^84,85^. A similar case for hair – a distantly related keratinous matrix – remains elusive however. A recent investigation using radiolabeled GCs in monkeys has instead presented fairly conclusive evidence that GCs do not form discrete bands in strands of hair, but that GCs move along the shaft of hair post-deposition^86^. The difference may lie in the gauge and density of the matrices, with baleen samples being extracted from a depth of several centimeters, using power tools, as opposed to processing the entirety of a micrometer-thick hair.

Regardless, the evidence provided by sub-sectioning of hairs, taken altogether, rather seems to suggest that hGCs are distributed along a strand of hair by longitudinal transport of the hydrophobic hormones, whether through diffusion or capillary action (possibly helped along by the waxy sebum). Reading too much into point-to-point fluctuations thus currently appears to be a case of seeing patterns where there are none to be found.

## Conclusions

Combining results of controlled studies with the correlational evidence, it seems fair to state that hGC levels seem to relate to central HPA axis functioning. GC levels in hairs appear to be an appropriate marker of *ongoing* physiological stress. If the stressor persists, hGC analyses will remain useful; however, it is currently unadvisable to interpret events in the past based on hGC levels. The idea of GCs being locked into place, providing a historical record of HPA axis functioning has been called into question every time it has been tested in a controlled experiment. Based on the collected evidence we would strongly advise against sub-segmenting hairs, speculating about specific periods in the past. We would be delighted to be proven wrong by a future study, but there is something to be said about, not only the studies our search strategy uncovered, but also the ones that could not be found. Whereas it is hard to design a study where subjects’ stress levels are controlled for weeks on end, it is far from impossible to design a study to test the hypothesis that a stressor in the past can be uncovered in a specific segment of hair. Yet, these studies are nowhere to be found. Whereas the data material did not allow for a stringent exploration of publication bias, it seems highly probable that a number of studies providing negative results have remained unpublished. With this review, and others like it, it is our hope that these negative findings may find their way into publications, providing a better picture of when hGC analyses are appropriate, and when they are not.

We strongly recommend that current and future research into hGC analyses focus on some of the fundamental questions. How are GCs incorporated into hairs? How long do they remain post-deposition? There are a number of basic questions that can be answered by small means, utilizing clever study design. With two notable exceptions^9,86^, studies utilizing radioisotope-labelled GCs are virtually completely missing; yet, the information that could be gained from studies of this type is invaluable. Applied studies utilizing hGC analyses, in the meanwhile, would do well to approach theoretical concepts surrounding hGCs in an agnostic fashion. We do not currently know how far into the past we can measure stress through sampling hairs. Stating that a certain protocol measures e.g. three months of preceding stress is misleading and perpetuates misinformation. Moreover, it is important that the duration of stressors be recorded and reported as accurately as possible. If we are able to pin down the temporality of hGCs as a stress marker, findings of studies in the past may have to be re-interpreted. This will however only be possible if there is enough information on timing of stressors relative to hair samplings. By detailing, as best as they can, the timing of stressors, researchers will, in a sense, future-proof their study results. Moreover, we hope that researchers will be more wary of unaccounted-for sources of stress in their studies. In many cases, these cannot be avoided. Consequently, we would encourage the reporting of possible “contaminating factors” – sources of stress that could not be accounted for in the study design. We would also urge authors to publish their raw data, or, at the very least, keep a record of them. In our investigation we were disappointed to find peer-reviewed publications missing crucial basic information (e.g. the number of subjects analyzed in a study) and to learn, when contacting the authors, that the information could not be produced. With most journals being able to host supporting data files online, and a host of repositories available for when journals cannot, there is no reason for not making data available and risking losing important records. Finally, we will note that many of the analyzed papers utilizing hGC analyses have made useful contributions to science; whether giving a voice to overlooked wildlife, trying to improve animal welfare, or assessing the mental wellbeing of people. We may seem critical of some of these studies, however, this comes from an adamant belief that we can and should do even better. We must constantly hold ourselves to a higher standard, in order to improve our field of research.

## Supplementary information

Supplemental Materials A (material & methods)

Supplemental Materials B (additional results)

Supplemental Materials C (pre-registered protocol)

Dataset

## References

[1] Davenport MD, Tiefenbacher S, Lutz CK, Novak MA, Meyer JS (2006). Analysis of endogenous cortisol concentrations in the hair of rhesus macaques. Gen. Comp. Endocrinol..

[2] Koren L (2002). A novel method using hair for determining hormonal levels in wildlife. Anim. Behav..

[3] Wosu AC, Valdimarsdóttir U, Shields AE, Williams DR, Williams MA (2013). Correlates of cortisol in human hair: implications for epidemiologic studies on health effects of chronic stress. Ann. Epidemiol..

[4] Luo H (2012). Hair cortisol level as a biomarker for altered hypothalamic-pituitary-adrenal activity in female adolescents with posttraumatic stress disorder after the 2008 Wenchuan earthquake. Biological Psychiatry.

[5] Carlitz EH, Kirschbaum C, Stalder T, van Schaik CP (2014). Hair as a long-term retrospective cortisol calendar in orang-utans (Pongo spp.): New perspectives for stress monitoring in captive management and conservation. Gen. Comp. Endocrinol..

[6] Loerbroks A, Hoffmann F, Grimm A, Kirschbaum C (2011). Stressful ancient Egypt? Assessing cortisol concentrations in a Mummy’s hair. Psychosomatic Medicine.

[7] Ito N (2005). Human hair follicles display a functional equivalent of the hypothalamic-pituitary-adrenal axis and synthesize cortisol. The FASEB journal.

[8] Salaberger T (2016). Influence of external factors on hair cortisol concentrations. Gen. Comp. Endocrinol..

[9] Keckeis K (2012). Hair cortisol: a parameter of chronic stress? Insights from a radiometabolism study in guinea pigs. Journal of Comparative Physiology B.

[10] Heimbürge S, Kanitz E, Otten W (2019). The use of hair cortisol for the assessment of stress in animals. Gen. Comp. Endocrinol..

[11] Sharpley CF, McFarlane JR, Slominski A (2012). Stress-linked cortisol concentrations in hair: what we know and what we need to know. Reviews in the Neurosciences.

[12] Sharpley CF, Kauter KG, McFarlane JR (2010). Diurnal variation in peripheral (hair) vs central (saliva) HPA axis cortisol concentrations. Clinical medicine insights. Endocrinology and diabetes.

[13] Sharpley CF, Kauter KG, McFarlane JR (2009). An Initial Exploration of *in vivo* Hair Cortisol Responses to a Brief Pain Stressor: Latency, Localization and Independence Effects. Physiological Research.

[14] Cattet M (2014). Quantifying long-term stress in brown bears with the hair cortisol concentration: a biomarker that may be confounded by rapid changes in response to capture and handling. Conservation Physiology.

[15] Sachs H (1997). History of hair analysis. Forensic science international.

[16] Gaillard Y, Vayssette F, Pépin G (2000). Compared interest between hair analysis and urinalysis in doping controls: Results for amphetamines, corticosteroids and anabolic steroids in racing cyclists. Forensic science international.

[17] Stokes, S. In *Velonation* (2013).

[18] Cirimele V, Kintz P, Dumestre V, Goulle J, Ludes B (2000). Identification of ten corticosteroids in human hair by liquid chromatography–ionspray mass spectrometry. Forensic science international.

[19] Yamada J, Stevens B, de Silva N, Klein J, Koren G (2003). Hair cortisol as a biologic marker of chronic stress in neonates: A pilot study. Pediatric Research.

[20] Yamada J (2007). Hair cortisol as a potential biologic marker of chronic stress in hospitalized neonates. Neonatology.

[21] Kicman AT, Cowan DA (2009). Subject‐based profiling for the detection of testosterone administration in sport. Drug Test. Anal..

[22] LeBeau MA, Montgomery MA, Brewer JD (2011). The role of variations in growth rate and sample collection on interpreting results of segmental analyses of hair. Forensic science international.

[23] Sachs H (1995). Theoretical limits of the evaluation of drug concentrations in hair due to irregular hair growth. Forensic science international.

[24] Mieczkowski T, Newel R (2000). Statistical examination of hair color as a potential biasing factor in hair analysis. Forensic science international.

[25] Wennig R (2000). Potential problems with the interpretation of hair analysis results. Forensic science international.

[26] Staufenbiel SM, Penninx BW, Spijker AT, Elzinga BM, van Rossum EF (2013). Hair cortisol, stress exposure, and mental health in humans: a systematic review. Psychoneuroendocrinology.

[27] Karlén J, Ludvigsson J, Frostell A, Theodorsson E, Faresjö T (2011). Cortisol in hair measured in young adults-a biomarker of major life stressors?. BMC clinical pathology.

[28] Hinkelmann K (2013). Association between childhood trauma and low hair cortisol in depressed patients and healthy control subjects. Biol Psychiatry.

[29] Grassi-Oliveira R (2012). Hair cortisol and stressful life events retrospective assessment in crack cocaine users. The American journal of drug and alcohol abuse.

[30] Steudte S (2011). Increased cortisol concentrations in hair of severely traumatized Ugandan individuals with PTSD. Psychoneuroendocrinology.

[31] Steudte S (2013). Hair Cortisol as a Biomarker of Traumatization in Healthy Individuals and Posttraumatic Stress Disorder Patients. Biological Psychiatry.

[32] Vaghri Z (2013). Hair cortisol reflects socio-economic factors and hair zinc in preschoolers. Psychoneuroendocrinology.

[33] Mastromonaco GF, Gunn K, McCurdy-Adams H, Edwards D, Schulte-Hostedde AI (2014). Validation and use of hair cortisol as a measure of chronic stress in eastern chipmunks (Tamias striatus). Conservation Physiology.

[34] Ashley N (2011). Glucocorticosteroid concentrations in feces and hair of captive caribou and reindeer following adrenocorticotropic hormone challenge. Gen. Comp. Endocrinol..

[35] Dettmer AM, Novak MA, Suomi SJ, Meyer JS (2012). Physiological and behavioral adaptation to relocation stress in differentially reared rhesus monkeys: Hair cortisol as a biomarker for anxiety-related responses. Psychoneuroendocrinology.

[36] Scorrano, F. *et al*. Validation of the long-term assessment of hypothalamic–pituitary–adrenal activity in rats using hair corticosterone as a biomarker. *The FASEB Journal*, fj. 14-254474 (2014). 10.1096/fj.14-254474 25398766

[37] Herane Vives A (2015). The relationship between cortisol, stress and psychiatric illness: New insights using hair analysis. Journal of Psychiatric Research.

[38] Stalder T (2017). Stress-related and basic determinants of hair cortisol in humans: a meta-analysis. Psychoneuroendocrinology.

[39] Kilkenny C, Browne WJ, Cuthill IC, Emerson M, Altman DG (2010). Improving bioscience research reporting: the ARRIVE guidelines for reporting animal research. PLoS Biol..

[40] Russell E (2015). Toward Standardization of Hair Cortisol Measurement: Results of the First International Interlaboratory Round Robin. Therapeutic Drug Monitoring.

[41] Gow R, Thomson S, Rieder M, Van Uum S, Koren G (2010). An assessment of cortisol analysis in hair and its clinical applications. Forensic science international.

[42] Cook NJ (2012). Review: Minimally invasive sampling media and the measurement of corticosteroids as biomarkers of stress in animals. Canadian Journal of Animal Science.

[43] Bryan HM, Darimont CT, Paquet PC, Wynne-Edwards KE, Smits JEG (2013). Stress and Reproductive Hormones in Grizzly Bears Reflect Nutritional Benefits and Social Consequences of a Salmon Foraging Niche. PLoS ONE.

[44] Moya D, Schwartzkopf-Genswein KS, Veira DM (2013). Standardization of a non-invasive methodology to measure cortisol in hair of beef cattle. Livestock Science.

[45] Moya D (2015). Effect of grain type and processing index on growth performance, carcass quality, feeding behavior, and stress response of feedlot steers. Journal of Animal Science.

[46] Terwissen CV, Mastromonaco GF, Murray DL (2013). Influence of adrenocorticotrophin hormone challenge and external factors (age, sex, and body region) on hair cortisol concentration in Canada lynx (Lynx canadensis). General and Comparative Endocrinology.

[47] Neumann A (2017). Predicting hair cortisol levels with hair pigmentation genes: a possible hair pigmentation bias. Scientific reports.

[48] Manenschijn L, Van Kruysbergen RGPM, De Jong FH, Koper JW, Van Rossum EFC (2011). Shift work at young age is associated with elevated long-term cortisol levels and body mass index. Journal of Clinical Endocrinology and Metabolism.

[49] Qin D (2015). Cortisol responses to chronic stress in adult macaques: moderation by a polymorphism in the serotonin transporter gene. Behav Brain Res.

[50] Kamps AWA (2014). Children with asthma have significantly lower long-term cortisol levels in their scalp hair than healthy children. Acta Paediatrica.

[51] Yu T (2015). Determination of endogenous corticosterone in rodent’s blood, brain and hair with LC-APCI-MS/MS. Journal of Chromatography B-Analytical Technologies in the Biomedical and Life Sciences.

[52] Accorsi PA (2008). Cortisol determination in hair and faeces from domestic cats and dogs. General and Comparative Endocrinology.

[53] D’Anna-Hernandez KL, Ross RG, Natvig CL, Laudenslager ML (2011). Hair cortisol levels as a retrospective marker of hypothalamic–pituitary axis activity throughout pregnancy: comparison to salivary cortisol. Physiology & behavior.

[54] Sauve B, Koren G, Walsh G, Tokmakejian S, Van Uum SHM (2007). Measurement of cortisol in human hair as a biomarker of systemic exposure. Clin Invest Med.

[55] Simmons JP, Nelson LD, Simonsohn U (2011). False-positive psychology: Undisclosed flexibility in data collection and analysis allows presenting anything as significant. Psychol Sci.

[56] Gelman, A. & Loken, E. The garden of forking paths: Why multiple comparisons can be a problem, even when there is no “fishing expedition” or “p-hacking” and the research hypothesis was posited ahead of time. Department of Statistics, Columbia University (2013).

[57] Pulopulos MM (2014). Hair cortisol and cognitive performance in healthy older people. Psychoneuroendocrinology.

[58] Schalinski Inga, Elbert Thomas, Steudte-Schmiedgen Susann, Kirschbaum Clemens (2015). The Cortisol Paradox of Trauma-Related Disorders: Lower Phasic Responses but Higher Tonic Levels of Cortisol Are Associated with Sexual Abuse in Childhood. PLOS ONE.

[59] Vanaelst B (2012). Intercorrelations between serum, salivary, and hair cortisol and child-reported estimates of stress in elementary school girls. Psychophysiology.

[60] Russell E, Koren G, Rieder M, Van Uum S (2012). Hair cortisol as a biological marker of chronic stress: current status, future directions and unanswered questions. Psychoneuroendocrinology.

[61] Stalder T, Kirschbaum C (2012). Analysis of cortisol in hair–State of the art and future directions. Brain, behavior, and immunity.

[62] Brearley G, McAlpine C, Bell S, Bradley A (2012). Influence of urban edges on stress in an arboreal mammal: a case study of squirrel gliders in southeast Queensland, Australia. Landscape ecology.

[63] Roth LS, Faresjo A, Theodorsson E, Jensen P (2016). Hair cortisol varies with season and lifestyle and relates to human interactions in German shepherd dogs. Sci Rep.

[64] Henley P (2014). Cultural and socio-economic conditions as factors contributing to chronic stress in sub-Saharan African communities. Canadian Journal of Physiology and Pharmacology.

[65] Gidlow CJ, Randall J, Gillman J, Smith GR, Jones MV (2016). Natural environments and chronic stress measured by hair cortisol. Landscape and Urban Planning.

[66] Natt, D., Johansson, I., Faresjo, T., Ludvigsson, J. & Thorsell, A. High cortisol in 5-year-old children causes loss of DNA methylation in SINE retrotransposons: a possible role for ZNF263 in stress-related diseases. *Clinical Epigenetics* **7**, 10.1186/s13148-015-0123-z (2015). 10.1186/s13148-015-0123-z PMC4559301 26339299

[67] Gonzalez-de-la-Vara MD (2011). Effects of adrenocorticotropic hormone challenge and age on hair cortisol concentrations in dairy cattle. Canadian Journal of Veterinary Research-Revue Canadienne De Recherche Veterinaire.

[68] Jarcho MR, Massner KJ, Eggert AR, Wichelt EL (2016). Behavioral and physiological response to onset and termination of social instability in female mice. Horm Behav.

[69] Bryan HM, Adams AG, Invik RM, Wynne-Edwards KE, Smits JE (2013). Hair as a meaningful measure of baseline cortisol levels over time in dogs. Journal of the American Association for Laboratory Animal Science.

[70] Chan J, Sauvé B, Tokmakejian S, Koren G, Van Uum S (2014). Measurement of cortisol and testosterone in hair of obese and non-obese human subjects. Experimental and clinical endocrinology & diabetes: official journal, German Society of Endocrinology [and] German Diabetes Association.

[71] Lightman SL, Conway-Campbell BL (2010). The crucial role of pulsatile activity of the HPA axis for continuous dynamic equilibration. Nat. Rev. Neurosci..

[72] Whitten P, Brockman D, Stavisky R (1998). Recent advances in noninvasive techniques to monitor hormone‐behavior interactions. Am J Phys Anthropol.

[73] Boesch M (2015). Hair cortisol concentration is unaffected by basic military training, but related to sociodemographic and environmental factors. Stress.

[74] Gidlow CJ, Randall J, Gillman J, Silk S, Jones MV (2016). Hair cortisol and self-reported stress in healthy, working adults. Psychoneuroendocrinology.

[75] Campbell J, Ehlert U (2012). Acute psychosocial stress: does the emotional stress response correspond with physiological responses?. Psychoneuroendocrinology.

[76] Gao W (2014). Temporal features of elevated hair cortisol among earthquake survivors. Psychophysiology.

[77] Heinze K, Lin A, Reniers RL, Wood SJ (2016). Longer-term increased cortisol levels in young people with mental health problems. Psychiatry Res.

[78] Steudte-Schmiedgen S (2015). Hair cortisol concentrations and cortisol stress reactivity predict PTSD symptom increase after trauma exposure during military deployment. Psychoneuroendocrinology.

[79] Chu X-X (2014). A natural model of behavioral depression in postpartum adult female cynomolgus monkeys (Macaca fascicularis). Zoological Research.

[80] Klumbies Elisabeth, Braeuer David, Hoyer Juergen, Kirschbaum Clemens (2014). The Reaction to Social Stress in Social Phobia: Discordance between Physiological and Subjective Parameters. PLoS ONE.

[81] Kapoor, A., Lubach, G. R., Ziegler, T. E. & Coe, C. L. Hormone levels in neonatal hair reflect prior maternal stress exposure during pregnancy. *Psychoneuroendocrinology* (2016). 10.1016/j.psyneuen.2016.01.010 PMC4788554 26802598

[82] Skoluda N, Dettenborn L, Stalder T, Kirschbaum C (2012). Elevated hair cortisol concentrations in endurance athletes. Psychoneuroendocrinology.

[83] Dettenborn L, Tietze A, Bruckner F, Kirschbaum C (2010). Higher cortisol content in hair among long-term unemployed individuals compared to controls. Psychoneuroendocrinology.

[84] Hunt K. E., Stimmelmayr R., George C., Hanns C., Suydam R., Brower H., Rolland R. M. (2014). Baleen hormones: a novel tool for retrospective assessment of stress and reproduction in bowhead whales (Balaena mysticetus). Conservation Physiology.

[85] Hunt KE, Lysiak NS, Moore M, Rolland RM (2017). Multi-year longitudinal profiles of cortisol and corticosterone recovered from baleen of North Atlantic right whales (Eubalaena glacialis). Gen. Comp. Endocrinol..

[86] Kapoor A, Schultz-Darken N, Ziegler TE (2018). Radiolabel validation of cortisol in the hair of rhesus monkeys. Psychoneuroendocrinology.

[87] Moher D, Liberati A, Tetzlaff J, Altman DG, Group P (2009). Preferred reporting items for systematic reviews and meta-analyses: the PRISMA statement. PLoS Med..

