# Supplementary material for: A systematic review of studies utilizing hair glucocorticoids as a measure of stress suggests the marker is more appropriate for quantifying short-term stressors
Source: Sci Rep. 2019 Aug 19;9:11997. doi: 10.1038/s41598-019-48517-2 (PMC6701156; doi:10.1038/s41598-019-48517-2)
Supplement: Supplementary file 1 — Supplemental Materials A (material & methods) [file 41598_2019_48517_MOESM1_ESM.pdf]

Supplemental Materials A. Detailed material and methods

Supplement to:

A systematic review of studies utilizing hair glucocorticoids as a measure of stress suggests the marker is more appropriate for quantifying short-term stressors

Otto Kalliokoski[1], Finn K. Jellestad[2], Robert Murison[2]

[1] Department of Experimental Medicine, University of Copenhagen, Denmark

[2] Department of Biological and Medical Psychology, University of Bergen, Norway

Supplemental Materials A. Detailed material and methods

The following is a detailed account of the methods employed for the systematic review. The preregistered protocol, listing some of the methods below, has been included as Supplemental Materials C. Some changes were made underway and have been listed in the methods below.

1. Search strategy

Human and animal studies were retrieved through multiple electronic journal databases – Medline, Web of Science, EMBASE, Zoological Record, and PsycINFO (searches were performed January 13, 2016). The search strings were designed to be as broad as possible, ensuring that no relevant studies where hair glucocorticoids (hGCs) were used as a measure of stress, were excluded (search strings are listed in Table 1). Duplicate entries were removed and an initial title/abstract screening was carried out. Studies were removed from further analysis if all three reviewers independently flagged the entry as clearly irrelevant; i.e. the study included no hGC measurements. Subsequently, all papers citing the retained journal entries – identified using Google Scholar – were retrieved, duplicates were removed, and these additional studies were pooled with the original cohort.

Table 1. Exact search strings.
Note that the search strings are identical with the exception for slight differences in syntax demanded by the different search engines. Medical Subject Headings (MeSH) were explored, but ultimately excluded since they did not add hits to the already broad searches.

Journal databases	Search string used
Embase, Medline, and PsycINFO (accessed through Ovid)	*((hair OR fur OR coat OR "hair follicle*" OR whisker*) AND (cortisol OR corticostero* OR glucocortico* OR adrenocortic*) AND (stress* OR allosta* OR PTSD OR anxiety OR depress*))*
Web of Science Core Collection and Zoological Record (accessed through Web of Science)	*((“hair” OR “fur” OR “coat” OR "hair follicle*" OR whisker*) AND (“cortisol” OR corticostero* OR glucocortico* OR adrenocortic*) AND (stress* OR allosta* OR “PTSD” OR “anxiety” OR depress*))*

2. Inclusion/exclusion criteria

Only English-language peer-reviewed papers presenting original data were included for further analysis. Papers had to include quantification of glucocorticoids (GCs) in hair from a vertebrate species, used as a measure of physiological or psychological stress. Two designs were admissible: either hGCs were measured in a group of (purportedly) stressed individuals, related to a less stressed control group (or the same subjects sampled under a less stressful condition), or the measurements were related (correlated) to GC measurements in another biological matrix for the same individuals. Other biological matrices where GCs have previously been validated to be a measure of central HPA axis functioning/activity, are blood, saliva, urine and feces (for an overview, refer to e.g. Sheriff et al.[1]). We termed the investigations employing the former design “experimental studies” and the latter design “correlational studies.” Initial full-text screenings were carried out by three reviewers (the authors of the present report) independently; disagreements on whether to include a study were settled in a meeting where a consensus was reached for all studies without a previously unanimous decision.

3. Quality assessments

Quality assessments of data used in meta-analyses are crucial elements of a systematic review, and a preferred reporting item listed as part of the PRISMA statement[2]. Standardized checklists/protocols for quality assessments have been proposed for human[3,4] and animal[5,6] studies. To our knowledge, there are unfortunately no standardized schemes that can be used, unmodified, for both. Instead, we utilized the method guide developed by the Agency for Healthcare Research and Quality[7,8] as a foundation for our quality assessments, constructing a protocol for assessing external validity/reporting quality and a nine-point risk-of-bias checklist critically assessing internal validity (Table 2).

The latter checklist strove to highlight elements that could potentially bias the associated findings. Specifically, the checklist addressed the domains of selection bias, performance bias, attrition bias, detection bias and reporting bias. Experiments producing correlations were considered less likely to be influenced by biases (paired samples obtained from the same subject are internally consistent, even if external sources of stress are unaccounted for) and we deemed it difficult to determine sources of bias from their reporting. Consequently, we only applied our risk-of-bias checklist for the experimental studies.

Table 2. Risk of bias checklist. Each item was answered with a yes or no. When the paper did not provide sufficient information to answer the question or there was conflicting information, the item was marked as "unclear."

**Selection bias**
1. Were cases and controls selected appropriately?
2. Does the design or analysis control account for important confounding and modifying variables through matching, stratification, multivariable analysis, or other approaches?
**Performance bias**
3. Did researchers rule out any impact from a concurrent intervention or an unintended exposure that might bias results?
**Attrition bias**
4. Were missing data handled appropriately?
**Detection bias**
5. Were the cases and controls assessed concurrently under similar ambient conditions?
6. Were the outcome assessors blinded?
7. Were stressors assessed/defined using valid and reliable measures, implemented consistently across all study participants?
8. Were confounding variables assessed using valid and reliable measures, implemented consistently across all study participants?
**Reporting bias**
9. Are the results of all analyses reported (or data made available)?

4. Data extractions

Information on study design, including subject characteristics, was extracted along with basic data on sample treatment and analysis from the retained publications (Table 3). For correlational studies, the number of subjects were extracted together with a correlation coefficient. For studies where only the hypothesis test was presented, correlation coefficients were calculated using the p-value, where this was reported as an exact number (as opposed to a range, e.g. $p < 0.05$). For studies of a stress/control design, the number of subjects was extracted along with means and standard deviations (recalculated from other measures of dispersion, where needed) for the groups. Where values were only reported graphically, data were estimated using on-screen measuring software (Universal Desktop Ruler, AVPSoft). Where data had been transformed to conform to a normal distribution, the transformed values were extracted. If the measure of dispersion was unclear, we assumed the reported values were SEM, thereby producing a conservative estimate. Data were extracted by a non-blinded reviewer, checked independently by a second reviewer, and brought to a three-reviewer consensus meeting if the reviewers disagreed (the three reviewers are the authors of the report). If data were not extractable, or partially missing, the corresponding author for the study was contacted by email. Where a response could not be obtained after a reminder email, other methods were employed, including (but not limited to) contacting the first/last author of the publication, using ORCID to obtain up-to-date contact details for the corresponding author, and reaching out to authors over ResearchGate. If the missing data could not be

produced, or if an author did not respond despite multiple contact attempts over the course of one month, the study in question was removed.

Table 3. Form for extracting basic study characteristics. Questions for data extraction are provided with example categories for answers. Note that item 4 had to be recoded as the studies could not easily be slotted into the suggested categories (refer to text for details).

**Study characteristics**	
1. Study ID?	Authors, year, journal
2. What type of study is this?	Experimental/ correlational
3. What are the subjects?	Human (infants/adults/etc.), nonhuman animals (species?)
4. What type of stressor is studied?	Acute stressor/ intermittent stressor/ chronic stressor/PTSD/ Not applicable (correlational studies only)
**Subject selection**	
5. How many subjects were included in the study?	Numbers
6. Were the group sizes determined by an a priori power analysis according to the report?	Yes/no
**Subject information**	
7. What sex were the subjects?	Male/female/mixed/ unclear
8. What other subject characteristics are listed?	Age? Health conditions? Other characteristics?
9. For heterogeneous cohorts, do the researchers account for the heterogeneity in their tests?	Yes/no/unclear/not applicable (e.g. homogeneous groups)
**Sampling**	
11. Were multiple sites on the body sampled?	Yes/no/unclear
12. How were hairs sampled?	Whole hair with follicle/ whole hair without follicle/segment (lock) of hair/unclear
13. Were the hairs further segmented to create sub-samples?	Yes/no/unclear
**Sample processing**	
14. Were the hairs washed prior to extraction of GCs?	Yes/no/unclear
15. How were the hairs processed prior to extraction?	Cut/milled or mortared/ no processing/other
16. What extraction medium was used?	Methanol/ethanol/ propanol/other
**Analysis**	
17. What GCs were measured?	Cortisol/corticosterone/ other (e.g. relevant metabolites)
18. What analysis method was utilized?	Antibody-based (ELISA/EIA/RIA)/chromato graphy (GC/LC/HPLC)/ mass spectrometry (MS/HPLC-MS)/other

5. Data analysis

The extracted data material was expected to be highly heterogeneous as circulating GC concentrations are known to differ significantly between contexts, species, sexes, and subjects of differing ages[1,9-11]. Moreover, the

measured concentrations are dependent on the laboratory analyses (assays) employed[12,13] with different preferred ways of reporting further augmenting differences (e.g. some authors preferring to report the resulting concentration of the extraction medium, as opposed to the hair content of glucocorticoids). To address this heterogeneity, standardized mean differences were employed (specifically, we utilized Hedges' g) as the end-point comparison for the stress/control design studies. In order to not artificially inflate the weight of studies with multiple comparisons, best practices for combining study data were employed.

Where study designs employed multiple stressor groups, these were combined in accordance with the methods recommended by the Cochrane Collaboration[14] (for an explanation to why combining data is preferable to including multiple groups from the same study, refer to Figure 1). Specifically means (*x*) and standard deviations (s) for independent groups were merged as follows:

$$\bar{x} = \frac{\sum_{i=1}^{k} n_i \times x_i}{N} \qquad s = \sqrt{\frac{\sum_{i=1}^{k} (n_i - 1) s_i^2 + n_i (x_i - \bar{x})}{N - 1}}$$

The formulas in the Cochrane handbook have here been generalized for k groups (as opposed to the special case of two groups shown in Table 7.7.a).

Where multiple measurements were obtained from the same individual, these non-independent measurements were combined utilizing the methods outlined by Borenstein et al.[15]. For these repeated measure designs – where multiple hair samples were collected over time, or produced through segmentation of hairs – the non-independent nature of adjacent samples (i.e. neighboring time-points/hair segments) had to be considered. The means of related samples were merged similarly to the samples of unrelated subjects:

$$\bar{x} = \frac{\sum_{i=1}^{l} n_i \times x_i}{N}$$

When merging the standard deviations, the degree of inter-relatedness between samples needed to be considered. This was accomplished by including the average correlation between two sets of samples $r_{i,j}$. The average correlation was set to 0.75 (i.e.

Figure 1. A simple example of why combining data is preferable

We wish to include a study in our model that features two stress conditions – a high stress, and a low stress condition – that are both compared to the same control group:

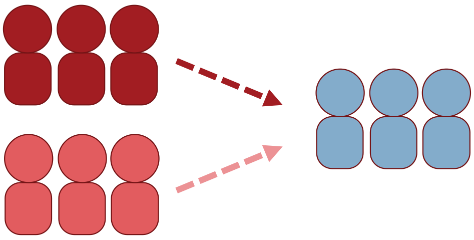

When including the two comparisons, separately, in our model, we artificially duplicate ("pseudo-replicate") the control group:

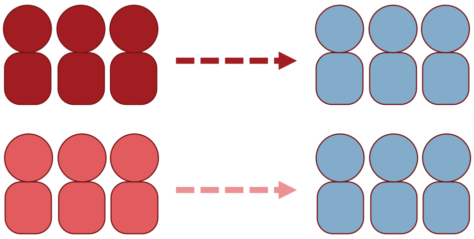

To avoid this scenario, we have instead chosen to merge measurements from the stress groups, forming a new combined stress group:

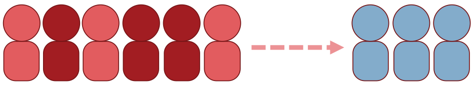

The drawback is that the comparison will now be a mix of the high and low stress conditions. However, since we are not attempting to establish the *magnitude* of any one particular stressor, but rather are attempting to find an overall trend/direction for all stress conditions, this is preferable for our analyses.

$r_{i,i+1} = 0.75$, $r_{i,i+2} = 0.75^2$, *etc.*) unless a correlation was explicitly stated in the paper. This estimate was based on raw data obtained from the analyzed studies (e.g. Schalinski et al.[16]) and robustness analysis[15]. Again, the generalized form for combining l inter-related sample sets has been derived (as opposed to the special case of two, shown by Borenstein et al.):

$$s = \frac{1}{N} \times \sqrt{\sum_{i=1}^{l} n_i^2 s_i^2 + 2 \times \sum_{i<j} r_{i,j} n_i n_j s_i s_j}$$

Due to the highly heterogeneous study designs and data material, the studies needed to be stratified to distinguish between types of stressor for a meaningful interpretation of data. Study designs were categorized as investigating induced (acute) stress, chronic stress, observed stress, self-assessed stress, past stress, or subjects suffering from PTSD. In the original protocol, comparisons were to be stratified by duration/temporality of the stressor (acute/intermittent/chronic), with PTSD studies analyzed separately. However, timing of the stressor (with respect to the subsequent sampling) proved hard to pin down with exactitude from the reporting. Instead of combining potentially incompatible study designs, the more granular subdivision was employed. Where we had originally hoped to rely on the authors' judgements to distinguish between acute and chronic stressors (and to possibly sort out intermittent stressors from the latter) the approach had to be rethought as stressors were seldom, if ever, classified in this manner. Instead, we chose to distinguish *induced* stressors, where the researchers themselves induced the stressful condition, from opportunistic sampling of stressful conditions of a *chronic* nature. The former set of studies would tend toward studying acute stressors, with studies employing ACTH injections and other protocols that would essentially "jolt" the HPA axis (e.g. capture of wild animals, stressful changes to housing of captive animals – man-made stressors, whether intentional or inadvertent). Some of the studies employed long intervals between induction of the first stressor and the last hair sampling, making the label "induced stress" more appropriate as these studies probably analyzed more than just an acute stress response. Samplings of populations exposed to stressful conditions – but where the researchers did not induce or have any control over the stressor, where stressfulness was simply inferred from context – were classified as chronic stress studies. In most, if not all, of these studies, the stressful condition had persisted for more than one month at the time of sampling and in some cases for the entirety of the subjects' lives. Categorizing these studies as "chronic stress" studies seemed appropriate given that other labels, such as natural, or spontaneous, would have brought connotations that were deemed to be inappropriate with respect to one or more of the included investigations (e.g. there is nothing natural about habitat destruction and nothing spontaneous about engaging in endurance sports). Stressors that could be verified to have had an effect, but also to have ended more than a week prior to hair sampling were separated out into their own category. To conform to the investigation by Stalder et al.[17], we termed these "*past stressors*." Models where stress was inferred on a subject-level, rather than from context, were similarly separated out from the chronic stressors. The timing and duration of these types of stress were hard to categorize with respect to the time of sampling. Here, the sampled population was often heterogeneous, and the experimenters relied on either self-reported measures, or on their own observations, to identify stressed individuals. With self-reported stress and observed stress being associated with different levels of accuracy, we chose to investigate these separately. Finally, PTSD studies were analyzed separately, in accordance with the pre-specified protocol. Whereas these findings are contentious, there are a number of studies reporting lowered levels of circulating GCs in PTSD patients[18]. This does not conform to the canonical view of an up-regulated HPA axis in relation to stressors. Thus, by necessity, studies of PTSD should be

analyzed separately. Despite the subdivision of data by study design, the stress models within each category were considered diverse enough to where data were synthesized using random effects models[19].

Correlation coefficients were synthesized by first transforming data onto Fisher's z scale, according to the method of Hedges and Olkin[20]. Random effects models were employed for each compartment separately, and where multiple coefficients were extracted from a single study, the weights of these z values were adjusted to avoid inflating the weight of any one study. For ease of interpretation, back-transformed data are presented.

Leave-one-out analysis[21] was employed to test whether the results of the meta-analyses could be considered robust or whether the overall conclusions could easily be influenced by moderate levels of publication bias. Originally, in the study protocol, funnel plot analysis and Egger regressions were suggested as a method for testing for publication bias. The highly heterogeneous data, which had to be stratified into subgroups to facilitate meaningful interpretation, were poorly suited for funnel plot analyses[22] however. Moreover it has been suggested that the use of standardized mean differences can lead to funnel plot asymmetry even when no publication bias exists[23]. Consequently, funnel plot analysis was not employed.

Analyses of experimental studies were conducted in JASP 0.9.0.1 (JASP Team), but since the software does not allow for adjusting weights of studies for summary estimates, correlation analyses were created in Microsoft Excel 2016 (Seattle WA, USA). Raw data files have been provided as supplemental material.

References

1 Sheriff, M. J., Dantzer, B., Delehanty, B., Palme, R. & Boonstra, R. Measuring stress in wildlife: techniques for quantifying glucocorticoids. *Oecologia.* **166**, 869-887 (2011).

2 Moher, D., Liberati, A., Tetzlaff, J., Altman, D. G. & Group, P. Preferred reporting items for systematic reviews and meta-analyses: the PRISMA statement. *PLoS Med.* **6**, e1000097 (2009).

3 Higgins, J. P. *et al.* The Cochrane Collaboration's tool for assessing risk of bias in randomised trials. *Bmj* **343**, d5928 (2011).

4 Downs, S. H. & Black, N. The feasibility of creating a checklist for the assessment of the methodological quality both of randomised and non-randomised studies of health care interventions. *J. Epidemiol. Community Health* **52**, 377-384 (1998).

5 Kilkenny, C., Browne, W. J., Cuthill, I. C., Emerson, M. & Altman, D. G. Improving bioscience research reporting: the ARRIVE guidelines for reporting animal research. *PLoS Biol.* **8**, e1000412 (2010).

6 Hooijmans, C. R. *et al.* SYRCLE's risk of bias tool for animal studies. *BMC Med. Res. Methodol.* **14**, 43 (2014).

7 Viswanathan, M. *et al.* Assessing the risk of bias of individual studies in systematic reviews of health care interventions. (2012).

8 Hartling, L. *et al.* Developing and testing a tool for the classification of study designs in systematic reviews of interventions and exposures. (2010).

9 Hansen, Å. M., Garde, A. H. & Persson, R. Sources of biological and methodological variation in salivary cortisol and their impact on measurement among healthy adults: a review. *Scand. J. Clin. Lab. Investig.* **68**, 448-458 (2008).

10 Koolhaas, J. M. *et al.* Stress revisited: a critical evaluation of the stress concept. *Neurosci. Biobehav. Rev.* **35**, 1291-1301 (2011).

11 Goymann, W. On the use of non-invasive hormone research in uncontrolled, natural environments: the problem with sex, diet, metabolic rate and the individual. *Methods Ecol. Evol.* **3**, 757-765 (2012).

12 Albar, W. F., Russell, E. W., Koren, G., Rieder, M. J. & Van Umm, S. H. Human hair cortisol analysis: comparison of the internationally-reported ELISA methods. *Clinical & Investigative Medicine* **36**, 312-316 (2013).

13 Russell, E. *et al.* Toward Standardization of Hair Cortisol Measurement: Results of the First International Interlaboratory Round Robin. *Therapeutic Drug Monitoring* **37**, 71-75 (2015).

14 Higgins, J. & Green, S. in *The Cochrane Collaboration* Ch. 7.7 Extracting study results and converting to the desired format, 170-181 (2011).

15 Borenstein, M., Hedges, L. V., Higgins, J. & Rothstein, H. R. in *Introduction to meta-analysis* 225-238 (2009).

16 Schalinski, I., Elbert, T., Steudte-Schmiedgen, S. & Kirschbaum, C. The Cortisol Paradox of Trauma-Related Disorders: Lower Phasic Responses but Higher Tonic Levels of Cortisol Are Associated with Sexual Abuse in Childhood. *Plos One* **10**, doi:10.1371/journal.pone.0136921 (2015).

17 Stalder, T. *et al.* Stress-related and basic determinants of hair cortisol in humans: a meta-analysis. *Psychoneuroendocrinology* **77**, 261-274 (2017).

18 Pan, X., Wang, Z., Wu, X., Wen, S. W. & Liu, A. Salivary cortisol in post-traumatic stress disorder: a systematic review and meta-analysis. *BMC Psychiatry* **18**, 324 (2018).

19 Borenstein, M., Hedges, L. V., Higgins, J. P. & Rothstein, H. R. A basic introduction to fixed-effect and random-effects models for meta-analysis. *Research synthesis methods* **1**, 97-111 (2010).

20 Hedges, L. V. O., Ingram. in *Statistical Methods for Meta-Analysis* Ch. 11, 223-246 (Academic Press, Inc., 1985).

21 Sutton, A. J. A., Keith R.; Jones, David R.; Sheldon, Trevor A.; Song, Fujian. in *Methods for Meta-analysis in Medical research Wiley Series in Probability and Statistics* (ed Noel A. C.; Fisher Cressie, Nicholas I.; Johnstone, Iain M.; Kadane, J. B.; Scott, David W.; Silverman, Bernard W.; Smith, Adrian F. M.; Teugels, Jozef L.) Ch. 9, 147-152 (John Wiley & Sons, Ltd., 2000).

22 Lau, J., Ioannidis, J. P., Terrin, N., Schmid, C. H. & Olkin, I. Evidence based medicine: The case of the misleading funnel plot. *BMJ* **333**, 597 (2006).

23 Zwetsloot, P.-P. *et al.* Standardized mean differences cause funnel plot distortion in publication bias assessments. *eLife* **6**, e24260 (2017).
