# Supplementary material for: A systematic review of studies utilizing hair glucocorticoids as a measure of stress suggests the marker is more appropriate for quantifying short-term stressors
Source: Sci Rep. 2019 Aug 19;9:11997. doi: 10.1038/s41598-019-48517-2 (PMC6701156; doi:10.1038/s41598-019-48517-2)
Supplement: Supplementary file 2 — Supplemental Materials B (additional results) [file 41598_2019_48517_MOESM2_ESM.pdf]

Supplemental Materials B. Additional results

Supplement to:

A systematic review of studies utilizing hair glucocorticoids as a measure of stress suggests the marker is more appropriate for quantifying short-term stressors

Otto Kalliokoski[1], Finn K. Jellestad[2], Robert Murison[2]

[1] Department of Experimental Medicine, University of Copenhagen, Denmark

[2] Department of Biological and Medical Psychology, University of Bergen, Norway

Supplemental materials B. Additional results

The central findings of the systematic review have been included in the main document (report). In the present document, additional results and raw data (/extracted data) have been included for full transparency. A full reference list including all the analyzed studies is appended at the end of this document.

1. Complete results from risk-of-bias analysis

Listed below are the final (post-consensus) results for the risk-of-bias analysis, shown in complete. This analysis is not meant to call into question any one study, but rather to analyze, in aggregate, the weight of the empirical evidence supporting the use of hGCs as a measure of stress. Notably, in no study are all of the sources of biases addressed, and in a few studies none are. Many of the checklist items are hard to address when conducting studies of chronic stressors in an uncontrolled environment (studying humans or other wildlife). The bottom-line is however that we cannot rule out a considerable influence of human bias in the aggregated data.

Entry	Risk of bias criterion								
	1	2	3	4	5	6	7	8	9
Ashley 2011	-	-	-	+	+	?	-	?	-
Boesch 2015	+	-	-	+	-	?	+	-	+
Bryan 2013	?	+	-	+	-	?	-	-	+
Bryan 2015	?	-	-	+	-	?	-	-	+
Carlitz 2014	?	-	-	-	-	?	-	-	-
Cattet 2014	+	+	-	+	-	?	+	+	+
Chu 2014	-	-	-	+	?	+	-	-	+
Corradini 2013	?	-	-	+	?	?	+	-	-
Davenport 2006	?	-	-	+	?	?	+	-	+
Dettenborn 2010	-	-	-	?	?	?	-	-	-
Dettmer 2014	-	-	-	?	-	?	-	-	-
Fairbanks 2011	+	+	+	-	-	?	+	?	-
Fourie 2015	-	-	-	?	-	?	+	-	+
Gao 2014	+	+	?	+	+	?	+	?	+
González-de-la-Vara 2011	+	+	?	+	+	?	+	+	+
Heinze 2016	?	-	?	+	?	?	+	-	+
Henley 2013	?	-	-	-	-	?	?	-	-
Jarcho 2016	+	+	+	+	+	?	?	+	-
Kapoor 2016	+	+	+	?	+	?	+	?	-
Karlén 2011	+	-	?	+	+	?	-	-	-
Klumbies 2014	?	-	-	?	+	?	+	?	+
Luo 2012	+	+	+	+	+	?	+	+	-
Manenschijn 2011	-	+	-	-	-	-	?	?	-
Mastromonaco 2014	?	-	-	+	?	?	+	?	-
Moya 2015	+	?	-	+	+	?	-	-	+
Nejad 2014	+	+	+	+	+	?	+	+	+
Oullette 2015	+	-	+	?	?	?	+	-	+
Qin 2015a	+	+	+	+	-	+	+	?	+
Schalinski 2015	?	+	?	?	+	?	+	+	+
Scorrano 2015	+	+	-	-	+	?	+	+	-
Skoluda 2012	-	-	-	?	?	?	?	+	?
Stalder 2014	-	+	+	+	?	?	+	+	+
Steudte 2013	+	-	-	+	+	?	+	+	+
Steudte-Schmiedgen 2015	+	+	-	+	-	?	+	+	-
Terwissen 2013	-	-	-	+	-	?	+	-	+
Van Uum 2008	+	-	+	+	?	?	?	+	-
Yamada 2007	?	?	-	+	?	?	?	+	-
Yu 2015	+	+	-	+	+	?	+	?	+

Checklist

Selection bias

1. Were cases and controls selected appropriately?
2. Does the design or analysis control account for important confounding and modifying variables through matching, stratification, multivariable analysis, or other approaches?

Performance bias

3. Did researchers rule out any impact from a concurrent intervention or an unintended exposure that might bias results?

Attrition bias

4. Were missing data handled appropriately?

Detection bias

5. Were the cases and controls assessed concurrently under similar ambient conditions?
6. Were the outcome assessors blinded?
7. Were stressors assessed/defined using valid and reliable measures, implemented consistently across all
8. Were confounding variables assessed using valid and reliable measures, implemented consistently across all study participants?

Reporting bias

9. Are the results of all analyses reported (or data made available)?

Key

+ Yes
? Unclear
- No

2. Sensitivity analysis using a leave-one-out approach

With the studied dataset parsed into subgroups, it was important to establish whether single studies could influence the synthesized results for the meta-analyses. A leave-one-out approach was taken to test the robustness of the presented forest plots. Listed below is the full set of analyses. The study exacting the largest influence on the end-results has been highlighted in bold type, demonstrating the effect of its removal. In most cases, removing this study lead to a decrease in between-studies heterogeneity (as demonstrated by a lower I^2 value) suggesting the removed study is the "odd man out" in the dataset.

2.1 Leave-one-out analysis of correlational studies

Correlations between hair and blood - random effects model

	Average correlation	95% CI	Subjects (n)	p	I^2
Overall	**0.357**	**0.076 - 0.585**	**237**	**0.014**	**75%**

Sensitivity analysis

Leave out:	Average correlation	95% CI	Subjects (n)	p	I^2
Chan 2014	0.426	0.079 - 0.681	180	0.018	79%
Corradini 2013	0.458	0.095 - 0.713	147	0.015	76%
Ouschan 2013	0.380	0.073 - 0.622	225	0.017	79%
Sauvé 2007	0.431	0.104 - 0.674	198	0.011	79%
Vanaelst 2012	0.386	0.068 - 0.633	218	0.018	79%
**Yu 2015**	**0.106**	**-0.032 - 0.240**	**217**	**0.131**	**0%**

Correlations between hair and saliva - random effects model

	Average correlation	95% CI	Subjects (n)	p	I^2
Overall	**0.232**	**0.116 - 0.341**	**687**	**1×10^{-4}**	**43%**

Sensitivity analysis

Leave out:	Average correlation	95% CI	Subjects (n)	p	I^2
Bennett 2010	0.209	0.093 - 0.320	645	5×10^{-4}	38%
Bryan 2013	0.241	0.129 - 0.347	680	4×10^{-5}	40%
Chan 2014	0.231	0.106 - 0.348	630	3×10^{-4}	44%
D'Anna-Hernandez 2011	0.221	0.102 - 0.334	666	3×10^{-4}	43%
**Davenport 2006**	**0.194**	**0.094 - 0.290**	**671**	**2×10^{-4}**	**26%**
Kuehl 2015	0.256	0.143 - 0.363	602	1×10^{-5}	35%
Manenschijn 2012	0.242	0.114 - 0.361	597	3×10^{-4}	45%
Moya 2013	0.222	0.097 - 0.340	666	6×10^{-4}	52%
Moya 2015	0.253	0.135 - 0.365	607	4×10^{-5}	39%
Pulopulos 2015	0.246	0.126 - 0.360	639	8×10^{-5}	43%
Sauvé 2007	0.228	0.105 - 0.344	648	3×10^{-4}	44%
Schalinski 2014	0.250	0.131 - 0.361	662	5×10^{-5}	44%
Steudte 2011	0.231	0.109 - 0.346	660	3×10^{-4}	45%
Steudte 2013	0.245	0.121 - 0.362	643	2×10^{-4}	47%
Van Holland 2012	0.221	0.101 - 0.335	658	4×10^{-4}	43%
Vanaelst 2012	0.222	0.100 - 0.338	654	4×10^{-4}	45%
Xie 2011	0.225	0.104 - 0.338	664	3×10^{-4}	44%

Correlations between hair and urine - fixed effects model

	Average correlation	95% CI	Subjects (n)	p	I^2
Overall	**0.132**	**-0.025 - 0.283**	**169**	**0.100**	**0%**

Sensitivity analysis

Leave out:	Average correlation	95% CI	Subjects (n)	p	I^2
Chan 2014	0.143	-0.056 - 0.330	112	0.158	3%
Chen 2014	0.129	-0.048 - 0.298	140	0.154	4%
Sauvé 2007	0.067	-0.113 - 0.243	130	0.464	0%
**Sumra 2015**	**0.173**	**0.000 - 0.336**	**138**	**0.050**	**0%**
Wippert 2014	0.142	-0.020 - 0.297	156	0.087	0%

Correlations between hair and feces - random effects model

	Average correlation	95% CI	Subjects (n)	p	I^2
Overall	**0.556**	**0.205 - 0.780**	**172**	**0.003**	**83%**

Sensitivity analysis

Leave out:	Average correlation	95% CI	Subjects (n)	p	I^2
**Accorsi 2008**	**0.330**	**0.05 - 0.558**	**116**	**0.020**	**38%**
Bryan 2013	0.537	0.147 - 0.782	165	0.009	85%
Mastromonaco 2014	0.608	0.231 - 0.826	110	0.003	79%
Tallo-Parra 2013	0.573	0.188 - 0.805	155	0.006	84%
Yamanashi 2013	0.595	0.226 - 0.814	163	0.003	85%
Moya 2013	0.597	0.204 - 0.825	151	0.005	84%

Comments:

The meta-correlation between GCs in blood and hGCs seems to be somewhat overestimated due to the extremely high correlations from the study by Yu et al. Removing them reduces both the synthesized correlation and the heterogeneity of the dataset (I^2 cannot even be estimated). By contrast, the meta-correlation between GCs in saliva and hGCs is constructed from a large enough dataset to be highly robust. By removing the study by Davenport et al. the heterogeneity can be reduced somewhat, but the synthesized correlation coefficient is only reduced marginally. The smallest dataset concerning the correlation between GCs in urine and hGCs is surprisingly robust. Removing the study by Sumra et al. produces a synthesized correlation coefficient that is statistically significant at the level of $p < 0.05$ (if only just). Similarly to the correlation with GCs in blood, the correlation between GCs in feces and hGC is reduced quite a bit if the study by Accorsi et al. is removed, while also reducing the dataset heterogeneity.

2.2 Leave-one-out analysis of experimental studies

Effect of induced/acute stress - random effects model

	Effect size summary	95% CI	Subjects (n)	p	I^2
Overall	**0.90**	**0.74 - 1.05**	**758**	**$< 10^{-5}$**	**0%**

Sensitivity analysis

Leave out:	Effect size summary	95% CI	Subjects (n)	p	I^2
Cattet 2014	0.94	0.78 - 1.10	716	$< 10^{-5}$	0%
Davenport 2006	0.89	0.73 - 1.04	736	$< 10^{-5}$	0%
**Fairbanks 2011**	**0.85**	**0.61 - 1.09**	**306**	**$< 10^{-5}$**	**0%**
González-de-la-Vara 2011	0.89	0.74 - 1.04	748	$< 10^{-5}$	0%
Jarcho 2016	0.90	0.75 - 1.06	710	$< 10^{-5}$	0%
Mastromonaco 2014	0.89	0.74 - 1.04	746	$< 10^{-5}$	0%
Moya 2015	0.90	0.74 - 1.05	718	$< 10^{-5}$	0%
Nejad 2013	0.90	0.75 - 1.06	731	$< 10^{-5}$	0%
Scorrano 2015	0.89	0.73 - 1.05	700	$< 10^{-5}$	0%
Terwissen 2013	0.89	0.74 - 1.05	752	$< 10^{-5}$	0%
Yu 2015	0.87	0.72 - 1.03	717	$< 10^{-5}$	0%

Effect of chronic/non-acute stress - random effects model

	Effect size summary	95% CI	Subjects (n)	p	I^2
Overall	**0.75**	**0.49 - 1.01**	**1730**	**$< 10^{-5}$**	**80%**

Sensitivity analysis

Leave out:	Effect size summary	95% CI	Subjects (n)	p	I^2
Boesch 2015	0.80	0.54 - 1.06	1448	$< 10^{-5}$	75%
Bryan 2013	0.81	0.55 - 1.07	1618	$< 10^{-5}$	78%
Bryan 2015	0.78	0.50 - 1.07	1552	$< 10^{-5}$	81%
Corradini 2013	0.73	0.46 -1.01	1668	$< 10^{-5}$	81%
Dettenborn 2010	0.77	0.49 - 1.04	1671	$< 10^{-5}$	82%
Dettmer 2014	0.70	0.44 - 0.96	1494	$< 10^{-5}$	77%
Fourie 2015	0.75	0.48 - 1.02	1684	$< 10^{-5}$	82%
Henley 2013	0.71	0.45 - 0.98	1658	$< 10^{-5}$	80%
Manenschijn 2011	0.78	0.50 - 1.06	1608	$< 10^{-5}$	81%
Qin 2015	0.69	0.45 - 0.94	1714	$< 10^{-5}$	78%
Skoluda 2012	0.76	0.47 - 1.06	1356	$< 10^{-5}$	81%
Stalder 2014	0.75	0.48 - 1.03	1690	$< 10^{-5}$	82%
**Van Uum 2008**	**0.68**	**0.43 - 0.93**	**1677**	**$< 10^{-5}$**	**78%**
Yamada 2007	0.75	0.48 - 1.03	1652	$< 10^{-5}$	82%

Effect of past stress - random effects model

	Effect size summary	95% CI	Subjects (n)	p	I^2
Overall	**-0.13**	**-0.80 - 0.53**	**95**	**0.69**	**60%**

Sensitivity analysis

Leave out:	Effect size summary	95% CI	Subjects (n)	p	I^2
Ashley 2011	-0.37	-1.07 - 0.32	35	0.29	N/A
Kapoor 2016	0.04	-1.15 - 1.23	60	0.95	78%

	**Effect of self-assessed stress - random effects model**				
	Effect size summary	95% CI	Subjects (n)	p	I^2
Overall	**0.18**	**-0.38 - 0.74**	**315**	**0.52**	**82%**

	**Sensitivity analysis**				
Leave out:	Effect size summary	95% CI	Subjects (n)	p	I^2
Gao 2014	0.02	-0.57 - 0.62	266	0.95	81%
Heinze 2016	0.09	-0.59 - 0.77	257	0.80	84%
Karlén 2011	0.10	-0.60 - 0.80	220	0.77	85%
Oullette 2015	0.32	-0.32 - 0.96	255	0.33	90%
**Steudte 2013**	**0.39**	**-0.12 - 0.90**	**262**	**0.13**	**73%**

	**Effect of externally assessed stress - random effects model**				
	Effect size summary	95% CI	Subjects (n)	p	I^2
Overall	**0.71**	**-0.27 - 1.70**	**296**	**0.15**	**90%**

	**Sensitivity analysis**				
Leave out:	Effect size summary	95% CI	Subjects (n)	p	I^2
**Carlitz 2014**	**0.32**	**-0.47 - 1.11**	**228**	**0.43**	**79%**
Chu 2014	0.96	-0.19 - 2.11	286	0.10	78%
Klumbies 2014	1.02	-0.02 - 2.05	130	0.05	80%
Luo 2012	0.59	-0.82 - 2.00	244	0.41	92%

	**Effect of PTSD - random effects model**				
	Effect size summary	95% CI	Subjects (n)	p	I^2
Overall	**-0.01**	**-0.52 - 0.51**	**398**	**0.98**	**78%**

	**Sensitivity analysis**				
Leave out:	Effect size summary	95% CI	Subjects (n)	p	I^2
Luo 2012	-0.12	-0.76 - 0.51	346	0.70	81%
Schalinski 2015	-0.18	-0.72 - 0.36	347	0.52	77%
**Steudte 2013**	**0.22**	**-0.27 - 0.71**	**345**	**0.38**	**68%**
Steudte-Schmiedgen 2015	0.06	-0.81 - 0.93	156	0.89	85%

Comments:

Both the effect of acute and chronic stressors on hGC concentrations seem to be robust, the summary effect being synthesized from a larger (> 10) number of studies, some of which are fairly large (with respect to subjects) in their own right. Removing any one study only has marginal effects. By contrast, the dataset concerning past stressors does not lend itself to a meaningful sensitivity analysis, with only three studies stemming from two publications. The studies investigating self-assessed stress make up a highly heterogeneous dataset. Removing any one study, however, does not change the conclusions of the meta-analysis: the comparison is not statistically significant. The externally assessed stressor studies are similarly heterogeneous. Removing either the study by Carlitz et al. or Klumbies et al. influence the summary effect size by roughly the same amount, only in opposite directions. Notably, however, the study by Carlitz et al. is

considerably smaller. Finally, the studies on hGC in PTSD is a set with effects pulling in both directions landing the summary estimate in the middle of the zero-effect line. Removing any one study only nudges the summary estimate marginally, providing no further clues with regards to directionality.

3. Data from correlational studies

Data extraction

Entry	Subjects	Correlation with GCs in	Comparison	Correlation coefficient	n	Comments
Accorsi 2008	Cats	Feces		0.902	27	

Entry	Subjects	Correlation with GCs in	Comparison	Correlation coefficient	n	Comments
Accorsi 2008	Dogs	Feces		0.67	29	

Entry	Subjects	Correlation with GCs in	Comparison	Correlation coefficient	n	Comments
Bennet 2010	Dogs	Saliva		0.48	42	

Entry	Subjects	Correlation with GCs in	Comparison	Correlation coefficient	n	Comments
Bryan 2013	Dogs	Feces		0.71	7	
		Saliva		-0.61	7	

Entry	Subjects	Correlation with GCs in	Comparison	Correlation coefficient	n	Comments
Chan 2014	Humans	Urine		0.11	57	
		Serum		0.16	57	
		Saliva		0.27	57	

Entry	Subjects	Correlation with GCs in	Comparison	Correlation coefficient	n	Comments
Chen 2014	Humans	Urine		0.144	29	ISM values used since the method was found to be superior.

Entry	Subjects	Correlation with GCs in	Comparison	Correlation coefficient	n	Comments
Corradini 2013	Dogs	Serum	Baseline	0.020	90	Correlation coefficients calculated from p-values and n.
			Post-ACTH	0.079	90	

Entry	Subjects	Correlation with GCs in	Comparison	Correlation coefficient	n	Comments
D'Anna-Hernandez 2011	Humans	Saliva	AUC	0.45	21	

Entry	Subjects	Correlation with GCs in	Comparison	Correlation coefficient	n	Comments
Davenport 2006	Monkeys	Saliva		0.797	16	

Entry	Subjects	Correlation with GCs in	Comparison	Correlation coefficient	n	Comments
Kuehl 2015	Humans	Saliva	CAR	-0.073	85	

Entry	Subjects	Correlation with GCs in	Comparison	Correlation coefficient	n	Comments
Manenschijn 2012	Humans	Saliva		0.16	90	

Entry	Subjects	Correlation with GCs in	Comparison	Correlation coefficient	n	Comments
Mastromonaco	Chipmunks	Feces		0.25	62	

Entry	Subjects	Correlation with GCs in	Comparison	Correlation coefficient	n	Comments
Moya 2013	Cattle	Saliva	Hair from hip	0.5264	21	Missing coefficients provided by Dr. Moya.
		Saliva	Hair from tail	0.6335	21	
		Saliva	Hair from head	0.423	21	
		Saliva	Hair from neck	0.312	21	
		Saliva	Hair from shoulder	-0.166	21	
		Feces	Hair from hip	0.048	21	
		Feces	Hair from tail	0.465	21	
		Feces	Hair from head	0.09	21	
		Feces	Hair from neck	0.458	21	
		Feces	Hair from shoulder	0.239	21	

Entry	Subjects	Correlation with GCs in	Comparison	Correlation coefficient	n	Comments
Moya 2015	Cattle	Saliva		-0.02	80	

Entry	Subjects	Correlation with GCs in	Comparison	Correlation coefficient	n	Comments
Ouschan 2013	Dogs	Blood	Post-ACTH	0.23	12	Correlation recreated from graph (using on-screen ruler).

Entry	Subjects	Correlation with GCs in	Comparison	Correlation coefficient	n	Comments
Pulopulos 2015	Humans	Saliva	Baseline	0.27	50	
		Saliva	AUC	-0.147	50	
		Saliva	Post-challenge	0.121	46	
		Saliva	Dynamic	0.237	46	

Entry	Subjects	Correlation with GCs in	Comparison	Correlation coefficient	n	Comments
Sauvé 2007	Humans	Saliva		0.306	39	
		Urine	24 h average	0.333	39	
		Serum		0.064	39	

Entry	Subjects	Correlation with GCs in	Comparison	Correlation coefficient	n	Comments
Schalinski 2014	Humans	Saliva	AUC vs. first segment	-0.14	28	
		Saliva	AUC vs. second segment	-0.11	21	
		Saliva	Dynamic vs. first segment	0.39	28	
		Saliva	Dynamic vs. second segment	0.24	21	

Entry	Subjects	Correlation with GCs in	Comparison	Correlation coefficient	n	Comments
Steudte 2011	Humans	Saliva	AUC	0.268	27	

Entry	Subjects	Correlation with GCs in	Comparison	Correlation coefficient	n	Comments
Steudte 2013	Humans	Saliva	Evening sample	0.085	44	
		Saliva	AUC	0.068	44	

Entry	Subjects	Correlation with GCs in	Comparison	Correlation coefficient	n	Comments
Sumra 2015	Humans	Urine	-0.058	31		Correlation constructed from raw data.

Entry	Subjects	Correlation with GCs in	Comparison	Correlation coefficient	n	Comments
Tallo-Parra 2015	Cattle	Feces	White hair	0.75926	17	Missing coefficient provided by Dr. Talló-Parra.
		Feces	Black hair	-0.03523	17	

Entry	Subjects	Correlation with GCs in	Comparison	Correlation coefficient	n	Comments
van Holland 2012	Humans	Saliva		0.41	29	

Entry	Subjects	Correlation with GCs in	Comparison	Correlation coefficient	n	Comments
Vanaelst 2012	Humans	Saliva	30 mins after waking	0.398	33	Missing coefficient provided by Dr. Michels.
		Saliva	AUC	0.398	32	
		Serum	Fasted sample	0.247	19	

Entry	Subjects	Correlation with GCs in	Comparison	Correlation coefficient	n	Comments
Wippert 2014	Humans	Urine	12 h, day one	0.13	13	

		Urine	12 h, day two	-0.16	13	

Entry	Subjects	Correlation with GCs in	Comparison	Correlation coefficient	n	Comments
Xie 2011	Humans	Saliva		0.383	23	Using average for 3 days.

Entry	Subjects	Correlation with GCs in	Comparison	Correlation coefficient	n	Comments
Yamanashi 2013	Chimpanzees	Feces		0.167	9	

Entry	Subjects	Correlation with GCs in	Comparison	Correlation coefficient	n	Comments
Yu 2015	Rats	Blood		0.803	12	

Entry	Subjects	Correlation with GCs in	Comparison	Correlation coefficient	n	Comments
Yu 2015	Mice	Serum		0.953	8	

4. Data extracted from experimental studies

Data extraction

Entry	Studied stressor	Classification	Subjects	Extraction method	Sample	Stress Mean	SD	n	Control Mean	SD	n	Additional notes
Ashley 2011	ACTH injections	Past stress	Caribou	Ruler	Neck, males[c]	5.17	2.71	5	4.40	1.81	5	
					Shoulder, males[c]	2.72	0.27	5	1.41	0.45	5	
					Rump, males[c]	1.71	0.38	5	1.45	0.45	5	
					Males[b]	2.27	0.31	5	2.42	0.78	5	
					Neck, females[c]	1.95	1.39	5	4.16	0.58	5	
					Shoulder, females[c]	1.27	0.13	5	2.43	0.27	5	
					Rump, females[c]	1.40	0.26	5	1.27	0.13	5	
					Females[b]	3.14	0.74	5	2.27	0.31	5	
					Males and females combined[a]	**3.17**	**1.28**	**10**	**2.35**	**0.95**	**10**	
Entry	Studied stressor	Classification	Subjects	Extraction method	Sample	Stress Mean	SD	n	Control Mean	SD	n	Additional notes
Ashley 2011	ACTH injections	Past stress	Reindeer	Ruler	Neck, males (2 IU)[c]	2.53	0.10	5	2.26	0.26	5	
					Shoulder, males (2 IU)[c]	3.02	0.10	5	3.10	0.42	5	
					Rump, males (2 IU)[c]	2.53	0.26	5	2.75	0.71	5	
					Males (2 IU)[b]	2.69	0.14	5	2.70	0.45	5	
					Neck, females (2 IU)[c]	2.56	0.26	5	2.26	0.10	5	
					Shoulder, females (2 IU)[c]	3.19	0.42	5	3.49	0.74	5	
					Rump, females (2 IU)[c]	2.00	0.10	5	3.01	0.42	5	
					Females (2 IU)[b]	2.58	0.25	5	2.92	0.44	5	
					Males and females combined (2 IU)[a]	2.64	0.59	10	2.82	0.80	10	
					Shoulder, males (8 IU)[c]	1.71	0.13	5	2.88	1.30	5	
					Shoulder, females (8 IU)[c]	1.48	0.10	5	3.13	0.94	5	
					Males and females combined (8 IU)[a]	1.60	0.55	10	3.01	1.60	10	
					Experiments combined[a]	**2.12**	**1.07**	**20**	**2.91**	**1.81**	**20**	
Entry	Studied stressor	Classification	Subjects	Extraction method	Sample	Stress Mean	SD	n	Control Mean	SD	n	Additional notes
Boesch 2015	Basic military training	Chronic stress	Humans	Text (table)	Scalp hairs[c]	**358.8**	**159.1**	**105**	**333.2**	**160.4**	**177**	
Entry	Studied stressor	Classification	Subjects	Extraction method	Sample	Stress Mean	SD	n	Control Mean	SD	n	Additional notes
Bryan 2013	Dietary stress	Chronic stress	Bears	Ruler	Hair snags, males[d]	-0.119	0.034	32	-0.119	0.035	55	
					Hair snags, females[d]	-0.114	0.023	10	-0.118	0.030	15	
					Males and females combined[a]	**-0.118**	**0.433**	**42**	**-0.119**	**0.416**	**70**	
Entry	Studied stressor	Classification	Subjects	Extraction method	Sample	Stress Mean	SD	n	Control Mean	SD	n	Additional notes
Bryan 2015	Hunting pressure	Chronic stress	Wolves	Ruler	Tundra vs. Forest[d]	2.78	0.30	103	2.63	0.47	45	
					Smoky[d]	2.97	0.60	30				
					High hunting pressure areas combined[a]	**2.82**	**0.40**	**133**	**2.63**	**0.47**	**45**	
Entry	Studied stressor	Classification	Subjects	Extraction method	Sample	Stress Mean	SD	n	Control Mean	SD	n	Additional notes
Carlitz 2014	Unspecified	Observed stress	Orangutans	Text	Various hair samples	**43.6**	**26.5**	**13**	**19.3**	**5.5**	**55**	
Entry	Studied stressor	Classification	Subjects	Extraction method	Sample	Stress Mean	SD	n	Control Mean	SD	n	Additional notes
Cattet 2014	Capture stress	Induced (acute) stress	Bears	Text	Hairs and hair snags[c]	**3.14**	**1.39**	**23**	**2.31**	**5.23**	**19**	Data in Figure 2 could not be extracted and has unknown numbers of subjects.
Entry	Studied stressor	Classification	Subjects	Extraction method	Sample	Stress Mean	SD	n	Control Mean	SD	n	Additional notes
Chu 2014	Post-partum depression	Observed stress	Monkeys	Text	Back hairs	**28.62**	**11.41**	**6**	**32.01**	**9.14**	**4**	
Entry	Studied stressor	Classification	Subjects	Extraction method	Sample	Stress Mean	SD	n	Control Mean	SD	n	Additional notes
Corradini 2013	Hypercortisolism	Chronic stress	Dogs	Data from authors	Chest hairs	**11.04**	**16.52**	**22**	**1.481**	**1.012**	**40**	SDs obtained from Dr. Fracassi
Entry	Studied stressor	Classification	Subjects	Extraction method	Sample	Stress Mean	SD	n	Control Mean	SD	n	Additional notes
Davenport 2006	Relocation stress	Induced (acute) stress	Monkeys	Text	Neck hairs[c]	**129.6**	**51.41**	**11**	**81.1**	**24.87**	**11**	
Entry	Studied stressor	Classification	Subjects	Extraction method	Sample	Stress Mean	SD	n	Control Mean	SD	n	Additional notes
Dettenborn 2010	Long-term unemployment	Chronic stress	Humans	Ruler	First hair segment	**22.09**	**15.64**	**31**	**15.29**	**6.51**	**28**	The sample n is unknown for the second hair segment
Entry	Studied stressor	Classification	Subjects	Extraction method	Sample	Stress Mean	SD	n	Control Mean	SD	n	Additional notes
Dettmer 2014	Crowding stress	Chronic stress	Monkeys	Text (table)	Males and females combined	**125.89**	**60.98**	**88**	**70.65**	**40.39**	**148**	
Entry	Studied stressor	Classification	Subjects	Extraction method	Sample	Stress Mean	SD	n	Control Mean	SD	n	Additional notes
Fairbanks 2011	Relocation stress	Induced (acute) stress	Monkeys	Ruler	Back hairs[c]	**67.15**	**17.98**	**226**	**52.92**	**12.29**	**226**	
Entry	Studied stressor	Classification	Subjects	Extraction method	Sample	Stress Mean	SD	n	Control Mean	SD	n	Additional notes
Fourie 2015	Anthropogenic disturbance	Chronic stress	Monkeys	Text	Back, males	189.1	54.3	6	115	34.1	13	Concentrations listed in ng/mg, but should probably be pg/mg. Unclear whether moderate human impact is to be considered stressful (omitted).
					Back, females	132.5	74.2	9	116.4	40.4	18	
					Males and females combined[a]	**161**	**64.3**	**15**	**116**	**37.3**	**31**	
Entry	Studied stressor	Classification	Subjects	Extraction method	Sample	Stress Mean	SD	n	Control Mean	SD	n	Additional notes
Gao 2014	Post-earthquake trauma	Self-assessed stress	Humans	Ruler	Time 1[d]	1.403	0.269	20	1.137	0.277	29	
					Time 2[d]	1.803	0.134	20	1.661	0.208	29	
					Time 3[d]	1.631	0.173	20	1.519	0.231	29	
					Time points combined[b]	**1.612**	**0.165**	**20**	**1.439**	**0.212**	**29**	
Entry	Studied stressor	Classification	Subjects	Extraction method	Sample	Stress Mean	SD	n	Control Mean	SD	n	Additional notes
González-de-la-Vara 2011	ACTH injections	Induced (acute) stress	Cattle	Ruler	Day 14	87.7	60.6	5	21.3	4.2	5	Using saline group as reference. Assuming SEM for error bars.
					Day 28	75.2	86.1	5	15.6	2.7	5	
					Day 44	16.0	2.5	5	19.2	3.4	5	
					Time points combined[b]	**59.6**	**46.6**	**5**	**18.7**	**3**	**5**	
Entry	Studied stressor	Classification	Subjects	Extraction method	Sample	Stress Mean	SD	n	Control Mean	SD	n	Additional notes
Heinze 2016	Mental health problems	Self-assessed stress	Humans	Ruler	1st segment	17.17	15.39	30	9.49	5.82	28	Using listed correlation coefficient (r = 0.286) for combinations.
					2nd segment	10.73	13.42	24	8.53	7.05	24	
					Segments combined[b]	**14.31**	**11.74**	**30**	**9.05**	**5.12**	**28**	
Entry	Studied stressor	Classification	Subjects	Extraction method	Sample	Stress Mean	SD	n	Control Mean	SD	n	Additional notes
Henley 2013	Socioeconomic stress	Chronic stress	Humans	Ruler	Scalp hairs	**181**	**59.5**	**40**	**112**	**54.6**	**32**	
Entry	Studied stressor	Classification	Subjects	Extraction method	Sample	Stress Mean	SD	n	Control Mean	SD	n	Additional notes
Jarcho 2016	Social instability stress	Induced (acute) stress	Mice	Ruler	Back hairs	**39.70**	**24.94**	**24**	**23.53**	**12.88**	**24**	
Entry	Studied stressor	Classification	Subjects	Extraction method	Sample	Stress Mean	SD	n	Control Mean	SD	n	Additional notes
Kapoor 2016	Acoustic startle stress	Past stress	Monkeys	Text (table)	Back hairs	97	30	22	110	40	13	Only data for mothers is used.
Entry	Studied stressor	Classification	Subjects	Extraction method	Sample	Stress Mean	SD	n	Control Mean	SD	n	Additional notes
Karlén 2011	"Serious life events"	Self-assessed stress	Humans	Text (table)	Scalp hairs	**32.98**	**56.90**	**20**	**16.32**	**22.53**	**75**	
Entry	Studied stressor	Classification	Subjects	Extraction method	Sample	Stress Mean	SD	n	Control Mean	SD	n	Additional notes
Klumbies 2014	Social phobia	Observed stress	Humans	Ruler	Scalp hairs	**6.64**	**16.14**	**88**	**6.47**	**15.83**	**78**	
Entry	Studied stressor	Classification	Subjects	Extraction method	Sample	Stress Mean	SD	n	Control Mean	SD	n	Additional notes
Luo 2012	Traumatic event	Observed stress	Humans	Ruler	Segment 1	6.38	1.77	32	5.96	1.40	20	Only segments 1-3 are used since authors state #4 should be unaffected. Assuming error bars are SEM, despite being listed as 95% CI.
					Segment 2	6.52	1.77	32	4.50	0.96	20	
					Segment 3	4.61	1.33	32	3.11	0.70	20	
					Segments combined[b]	**5.84**	**1.45**	**32**	**4.52**	**0.89**	**20**	
Entry	Studied stressor	Classification	Subjects	Extraction method	Sample	Stress Mean	SD	n	Control Mean	SD	n	Additional notes
Luo 2012	PTSD	PTSD	Humans	Ruler	Segment 1	5.33	1.44	32	5.96	1.40	20	
					Segment 2	5.23	1.44	32	4.50	0.96	20	
					Segment 3	4.30	1.22	32	3.11	0.70	20	
					Segments combined[b]	**4.95**	**1.22**	**32**	**4.52**	**0.89**	**20**	
Entry	Studied stressor	Classification	Subjects	Extraction method	Sample	Stress Mean	SD	n	Control Mean	SD	n	Additional notes
Manenschijn 2011	Shift work	Chronic stress	Humans	Text	Scalp hairs[d]	**1.675**	**0.520**	**33**	**1.473**	**0.520**	**89**	Assuming error bars are SEM, despite being listed as 95% CI.
Entry	Studied stressor	Classification	Subjects	Extraction method	Sample	Stress Mean	SD	n	Control Mean	SD	n	Additional notes
Mastromonaco 2014	ACTH injections	Induced (acute) stress	Chipmunks	Text and ruler	Leg hairs	**536.22**	**455.29**	**5**	**129.57**	**85.71**	**7**	The n's for the field study could not be obtained - only the first experiment is included. The average for the stress group has been recalculated to include two purported outliers.
Entry	Studied stressor	Classification	Subjects	Extraction method	Sample	Stress Mean	SD	n	Control Mean	SD	n	Additional notes
Moya 2015	Digestive problems	Induced (acute) stress	Cattle	Data from authors	Tail hairs	**2.06**	**0.425**	**20**	**1.69**	**0.421**	**20**	SDs obtained from Dr. Moya. Using only the highest and lowest stress groups, as the two middle groups are not clearly defined as being one or the other.
Entry	Studied stressor	Classification	Subjects	Extraction method	Sample	Stress Mean	SD	n	Control Mean	SD	n	Additional notes
Nejad 2013	Water restriction	Induced (acute) stress	Sheep	Text (table)	Short restriction	2.59	0.03	9	2.63	0.03	9	We assume that the RMSEs can be used for good estimates of the SDs.
					Long restriction	2.78	0.03	9				
					Water restrictions combined[a]	**2.69**	**0.10**	**18**	**2.63**	**0.03**	**9**	
Entry	Studied stressor	Classification	Subjects	Extraction method	Sample	Stress Mean	SD	n	Control Mean	SD	n	Additional notes
Oullette 2015	Psychosocial stress	Self-assessed stress	Humans	Text (table)	Scalp hairs	**126.67**	**165.38**	**30**	**244.58**	**449.00**	**30**	Only data for mothers is used.
Entry	Studied stressor	Classification	Subjects	Extraction method	Sample	Stress Mean	SD	n	Control Mean	SD	n	Additional notes

Qin 2015	Photoperiod manipulation	Chronic stress	Monkeys	Ruler	Back hairs[c]	**34.6**	**7.1**	**8**	**14.4**	**4.8**	**8**	
						Stress			Control			
Entry	Studied stressor	Classification	Subjects	Extraction method	Sample	Mean	SD	n	Mean	SD	n	Additional notes
Schalinski 2015	"Stress-related disorders"	PTSD	Humans	Data file (suppl. materials)	Non-CSA, segments combined[d]	1.84	0.57	19	1.66	0.62	12	Segments combined for each individual. Missing data completed through imputation.
					CSA, segments combined[d]	2.43	0.88	20				
					All traumatized subjects[a]	**2.14**	**0.79**	**39**	**1.66**	**0.62**	**12**	
						Stress			Control			
Entry	Studied stressor	Classification	Subjects	Extraction method	Sample	Mean	SD	n	Mean	SD	n	Additional notes
Scorrano 2015	Misc. stress protocols	Induced (acute) stress	Rats	Ruler	Experiment 2, IMO	18.8	4.9	6	10.2	3.2	6	
					Experiment 2, CUS	18.6	2.2	6				
					Stress groups combined[a]	18.7	3.6	12	10.2	3.2	6	
					Experiment 3	28.7	6.2	8	23.9	2.3	8	
					Experiment 4, low	49.5	28.0	8	15.5	7.1	8	
					Experiment 4 high	339.4	82.3	8				
					ACTH groups combined[a]	194.5	76.0	16	15.5	7.1	8	
					All stress groups combined[a]	**99.0**	**108.4**	**36**	**17.1**	**8.31**	**22**	
						Stress			Control			
Entry	Studied stressor	Classification	Subjects	Extraction method	Sample	Mean	SD	n	Mean	SD	n	Additional notes
Skoluda 2012	Intensive training	Chronic stress	Humans	Ruler	Scalp hairs	**18.18**	**8.53**	**304**	**12.43**	**5.56**	**70**	
						Stress			Control			
Entry	Studied stressor	Classification	Subjects	Extraction method	Sample	Mean	SD	n	Mean	SD	n	Additional notes
Stalder 2014	Caring for relative with dementia	Chronic stress	Humans	Text	Scalp hairs	**27.4**	**11.3**	**20**	**20.5**	**7.3**	**20**	
						Stress			Control			
Entry	Studied stressor	Classification	Subjects	Extraction method	Sample	Mean	SD	n	Mean	SD	n	Additional notes
Steudte 2013	Traumatic event	Self-assessed stress	Humans	Text	Scalp hairs	**7.78**	**7.01**	**25**	**15.75**	**14.77**	**28**	
						Stress			Control			
Entry	Studied stressor	Classification	Subjects	Extraction method	Sample	Mean	SD	n	Mean	SD	n	Additional notes
Steudte 2013	Traumatic event	PTSD	Humans	Text	Scalp hairs	**6.44**	**6.05**	**25**	**15.75**	**14.77**	**28**	
						Stress			Control			
Entry	Studied stressor	Classification	Subjects	Extraction method	Sample	Mean	SD	n	Mean	SD	n	Additional notes
Steudte-Schmiedgen 2015	Military deployment	PTSD	Humans	Text (table)	Scalp hairs	3.56	5.03	113	4.25	5.26	129	
						Stress			Control			
Entry	Studied stressor	Classification	Subjects	Extraction method	Sample	Mean	SD	n	Mean	SD	n	Additional notes
Terwissen 2013	ACTH injections	Induced (acute) stress	Lynxes	Ruler	Hairs[c]	**13.5**	**7.1**	**3**	**6.0**	**2.5**	**3**	
						Stress			Control			
Entry	Studied stressor	Classification	Subjects	Extraction method	Sample	Mean	SD	n	Mean	SD	n	Additional notes
van Uum 2008	Chronic pain	Chronic stress	Humans	Ruler	Scalp hairs[d]	**1.917**	**0.165**	**14**	**1.658**	**0.138**	**39**	Assumption: medians can be used as a good approximation of the mean by log-transforming data (implicitly assuming log-normal distribution of data).
						Stress			Control			
Entry	Studied stressor	Classification	Subjects	Extraction method	Sample	Mean	SD	n	Mean	SD	n	Additional notes
Yamada 2007	Neonatal illness	Chronic stress	Humans	Text	NICU vs. Control	**2.06**	**2.05**	**60**	**0.71**	**0.42**	**18**	
						Stress			Control			
Entry	Studied stressor	Classification	Subjects	Extraction method	Sample	Mean	SD	n	Mean	SD	n	Additional notes
Yu 2015	Aggression/social instability	Induced (acute) stress	Mice	Ruler	Back hairs	**71.9**	**45.1**	**11**	**38.1**	**19.5**	**8**	
						Stress			Control			
Entry	Studied stressor	Classification	Subjects	Extraction method	Sample	Mean	SD	n	Mean	SD	n	Additional notes
Yu 2015	Surgery/post-surgical pain	Induced (acute) stress	Rats	Text	Back hairs	**19.63**	**11**	**10**	**5.04**	**1.9**	**12**	

Legend

a) Independent samples combined according to Cochrane
b) Related samples combined according to Borenstein
c) Baseline values replacing control group
d) Transformed data (log/reciprocal/etc.) are used

5. Complete reference list of included publications

Listed below are the full references for the studies included in the analyses for the present study. Note that some publications will be listed in both categories.

5.1 References for experimental studies

Ashley, N. T., Barboza, P. S., Macbeth, B. J., Janz, D. M., Cattet, M. R. L., Booth, R. K., & Wasser, S. K. (2011). Glucocorticosteroid concentrations in feces and hair of captive caribou and reindeer following adrenocorticotropic hormone challenge. *General and comparative endocrinology, 172*(3), 382-391.

Boesch, M., Sefidan, S., Annen, H., Ehlert, U., Roos, L., Van Uum, S., . . . La Marca, R. (2015). Hair cortisol concentration is unaffected by basic military training, but related to sociodemographic and environmental factors. *Stress, 18*(1), 35-41.

Bryan, H. M., Darimont, C. T., Paquet, P. C., Wynne-Edwards, K. E., & Smits, J. E. G. (2013). Stress and Reproductive Hormones in Grizzly Bears Reflect Nutritional Benefits and Social Consequences of a Salmon Foraging Niche. *PloS one, 8*(11), e80537, 80531-80510.

Bryan, H. M., Smits, J. E., Koren, L., Paquet, P. C., Wynne-Edwards, K. E., & Musiani, M. (2015). Heavily hunted wolves have higher stress and reproductive steroids than wolves with lower hunting pressure. *Functional Ecology, 29*(3), 347-356.

Carlitz, E. H. D., Kirschbaum, C., Stalder, T., & van Schaik, C. P. (2014). Hair as a long-term retrospective cortisol calendar in orang-utans (Pongo spp.): New perspectives for stress monitoring in captive management and conservation. *General and comparative endocrinology, 195*, 151-156.

Cattet, M., Macbeth, B. J., Janz, D. M., Zedrosser, A., Swenson, J. E., Dumond, M., & Stenhouse, G. B. (2014). Quantifying long-term stress in brown bears with the hair cortisol concentration: a biomarker that may be confounded by rapid changes in response to capture and handling. *Conservation Physiology, 2*(1), cou026.

Chu, X.-X., Rizak, J. D., Yang, S.-C., Wang, J.-H., Ma, Y.-Y., & Hu, X.-T. (2014). A natural model of behavioral depression in postpartum adult female cynomolgus monkeys (Macaca fascicularis). *Zoological Research, 35*(3), 174-181.

Corradini, S., Accorsi, P. A., Boari, A., Beghelli, V., Mattioli, M., Famigli-Bergamini, P., & Fracassi, F. (2013). Evaluation of hair cortisol in the diagnosis of hypercortisolism in dogs. *J Vet Intern Med, 27*(5), 1268-1272.

Davenport, M. D., Tiefenbacher, S., Lutz, C. K., Novak, M. A., & Meyer, J. S. (2006). Analysis of endogenous cortisol concentrations in the hair of rhesus macaques. *General and comparative endocrinology, 147*(3), 255-261.

Dettenborn, L., Tietze, A., Bruckner, F., & Kirschbaum, C. (2010). Higher cortisol content in hair among long-term unemployed individuals compared to controls. *Psychoneuroendocrinology, 35*(9), 1404-1409.

Dettmer, A. M., Novak, M. A., Meyer, J. S., & Suomi, S. J. (2014). Population density-dependent hair cortisol concentrations in rhesus monkeys (Macaca mulatta). *Psychoneuroendocrinology, 42*, 59-67.

Fairbanks, L. A., Jorgensen, M. J., Bailey, J. N., Breidenthal, S. E., Grzywa, R., & Laudenslager, M. L. (2011). Heritability and genetic correlation of hair cortisol in vervet monkeys in low and higher stress environments. *Psychoneuroendocrinology, 36*(8), 1201-1208.

Fourie, N. H., Turner, T. R., Brown, J. L., Pampush, J. D., Lorenz, J. G., & Bernstein, R. M. (2015). Variation in vervet (Chlorocebus aethiops) hair cortisol concentrations reflects ecological disturbance by humans. *Primates, 56*(4), 365-373.

Gao, W., Zhong, P., Xie, Q., Wang, H., Jin, J., Deng, H., & Lu, Z. (2014). Temporal features of elevated hair cortisol among earthquake survivors. *Psychophysiology, 51*(4), 319-326.

Gonzalez-de-la-Vara, M. D., Valdez, R. A., Lemus-Ramirez, V., Vazquez-Chagoyan, J. C., Villa-Godoy, A., & Romano, M. C. (2011). Effects of adrenocorticotropic hormone challenge and age on hair cortisol concentrations in dairy cattle. *Canadian Journal of Veterinary Research-Revue Canadienne De Recherche Veterinaire, 75*(3), 216-221.

Heinze, K., Lin, A., Reniers, R. L., & Wood, S. J. (2016). Longer-term increased cortisol levels in young people with mental health problems. *Psychiatry Res, 236*, 98-104.

Henley, P., Jahedmotlagh, Z., Thomson, S., Hill, J., Darnell, R., Jacobs, D., . . . Koren, G. (2013). Hair Cortisol as a Biomarker of Stress Among a First Nation in Canada. *Therapeutic drug monitoring, 35*(5), 595-599.

Jarcho, M. R., Massner, K. J., Eggert, A. R., & Wichelt, E. L. (2016). Behavioral and physiological response to onset and termination of social instability in female mice. *Horm Behav, 78*, 135-140.

Kapoor, A., Lubach, G. R., Ziegler, T. E., & Coe, C. L. (2016). Hormone levels in neonatal hair reflect prior maternal stress exposure during pregnancy. *Psychoneuroendocrinology*.

Karlén, J., Ludvigsson, J., Frostell, A., Theodorsson, E., & Faresjö, T. (2011). Cortisol in hair measured in young adults-a biomarker of major life stressors? *BMC clinical pathology, 11*(1), 12.

Klumbies, E., Braeuer, D., Hoyer, J., & Kirschbaum, C. (2014). The Reaction to Social Stress in Social Phobia: Discordance between Physiological and Subjective Parameters. *PloS one, 9*(8).

Luo, H., Hu, X., Liu, X., Ma, X., Guo, W., Qiu, C., . . . Li, T. (2012). Hair cortisol level as a biomarker for altered hypothalamic-pituitary-adrenal activity in female adolescents with posttraumatic stress disorder after the 2008 Wenchuan earthquake. *Biological psychiatry, 72*(1), 65-69.

Manenschijn, L., Van Kruysbergen, R. G. P. M., De Jong, F. H., Koper, J. W., & Van Rossum, E. F. C. (2011). Shift work at young age is associated with elevated long-term cortisol levels and body mass index. *Journal of Clinical Endocrinology and Metabolism, 96*(11), E1862-E1865.

Mastromonaco, G. F., Gunn, K., McCurdy-Adams, H., Edwards, D., & Schulte-Hostedde, A. I. (2014). Validation and use of hair cortisol as a measure of chronic stress in eastern chipmunks (Tamias striatus). *Conservation Physiology, 2*(1), cou055.

Moya, D., He, M. L., Jin, L., Wang, Y., Penner, G. B., Schwartzkopf-Genswein, K. S., & McAllister, T. A. (2015). Effect of grain type and processing index on growth performance, carcass quality, feeding behavior, and stress response of feedlot steers. *Journal of animal science, 93*(6), 3091-3100.

Nejad, J. G., Lohakare, J. D., Son, J. K., Kwon, E. G., West, J. W., & Sung, K. I. (2014). Wool cortisol is a better indicator of stress than blood cortisol in ewes exposed to heat stress and water restriction. *animal, 8*(1), 128-132.

Ouellette, S. J., Russell, E., Kryski, K. R., Sheikh, H. I., Singh, S. M., Koren, G., & Hayden, E. P. (2015). Hair cortisol concentrations in higher- and lower-stress mother-daughter dyads: A pilot study of associations and moderators. *Dev Psychobiol, 57*(5), 519-534.

Qin, D., Chu, X., Feng, X., Li, Z., Yang, S., Lü, L., . . . Li, J. (2015). The first observation of seasonal affective disorder symptoms in Rhesus macaque. *Behavioural brain research, 292*, 463-469.

Schalinski, I., Elbert, T., Steudte-Schmiedgen, S., & Kirschbaum, C. (2015). The Cortisol Paradox of Trauma-Related Disorders: Lower Phasic Responses but Higher Tonic Levels of Cortisol Are Associated with Sexual Abuse in Childhood. *PloS one, 10*(8).

Scorrano, F., Carrasco, J., Pastor-Ciurana, J., Belda, X., Rami-Bastante, A., Bacci, M. L., & Armario, A. (2015). Validation of the long-term assessment of hypothalamic-pituitary-adrenal activity in rats using hair corticosterone as a biomarker. *Faseb Journal, 29*(3), 859-867.

Skoluda, N., Dettenborn, L., Stalder, T., & Kirschbaum, C. (2012). Elevated hair cortisol concentrations in endurance athletes. *Psychoneuroendocrinology, 37*(5), 611-617.

Stalder, T., Tietze, A., Steudte, S., Alexander, N., Dettenborn, L., & Kirschbaum, C. (2014). Elevated hair cortisol levels in chronically stressed dementia caregivers. *Psychoneuroendocrinology, 47*, 26-30.

Steudte, S., Kirschbaum, C., Gao, W., Alexander, N., Schonfeld, S., Hoyer, J., & Stalder, T. (2013). Hair Cortisol as a Biomarker of Traumatization in Healthy Individuals and Posttraumatic Stress Disorder Patients. *Biological psychiatry, 74*(9), 639-646.

Steudte-Schmiedgen, S., Stalder, T., Schonfeld, S., Wittchen, H.-U., Trautmann, S., Alexander, N., . . . Kirschbaum, C. (2015). Hair cortisol concentrations and cortisol stress reactivity predict PTSD symptom increase after trauma exposure during military deployment. *Psychoneuroendocrinology, 59*, 123-133.

Terwissen, C. V., Mastromonaco, G. F., & Murray, D. L. (2013). Influence of adrenocorticotrophin hormone challenge and external factors (age, sex, and body region) on hair cortisol concentration in Canada lynx (Lynx canadensis). *General and comparative endocrinology, 194*, 162-167.

Van Uum, S. H. M., Sauve, B., Fraser, L. A., Morley-Forster, P., Paul, T. L., & Koren, G. (2008). Elevated content of cortisol in hair of patients with severe chronic pain: A novel biomarker for stress. *Stress-the International Journal on the Biology of Stress, 11*(6), 483-488.

Yamada, J., Stevens, B., de Silva, N., Gibbins, S., Beyene, J., Taddio, A., . . . Koren, G. (2007). Hair cortisol as a potential biologic marker of chronic stress in hospitalized neonates. *Neonatology, 92*(1), 42-49.

Yu, T., Xu, H., Wang, W. W., Li, S. F., Chen, Z., & Deng, H. H. (2015). Determination of endogenous corticosterone in rodent's blood, brain and hair with LC-APCI-MS/MS. *Journal of Chromatography B-Analytical Technologies in the Biomedical and Life Sciences, 1002*, 267-276.

5.2 References for correlational studies

Accorsi, P. A., Carloni, E., Valsecchi, P., Viggiani, R., Garnberoni, M., Tarnanini, C., & Seren, E. (2008). Cortisol determination in hair and faeces from domestic cats and dogs. *General and comparative endocrinology, 155*(2), 398-402.

Bennett, A., & Hayssen, V. (2010). Measuring cortisol in hair and saliva from dogs: coat color and pigment differences. *Domestic animal endocrinology, 39*(3), 171-180.

Bryan, H. M., Adams, A. G., Invik, R. M., Wynne-Edwards, K. E., & Smits, J. E. (2013). Hair as a meaningful measure of baseline cortisol levels over time in dogs. *Journal of the American Association for Laboratory Animal Science, 52*(2), 189-196.

Chan, J., Sauvé, B., Tokmakejian, S., Koren, G., & Van Uum, S. (2014). Measurement of cortisol and testosterone in hair of obese and non-obese human subjects. *Experimental and clinical endocrinology & diabetes: official journal, German Society of Endocrinology [and] German Diabetes Association, 122*(6), 356-362.

Chen, Z., Li, J., Xu, G., Yang, J., Zhang, J., & Deng, H. (2014). Simultaneous measurements of cortisol and cortisone in urine and hair for the assessment of 11β-hydroxysteroid dehydrogenase activity among methadone maintenance treatment patients with LC-ESI–MS/MS. *Journal of Chromatography B, 969*, 77-84.

Corradini, S., Accorsi, P. A., Boari, A., Beghelli, V., Mattioli, M., Famigli-Bergamini, P., & Fracassi, F. (2013). Evaluation of hair cortisol in the diagnosis of hypercortisolism in dogs. *J Vet Intern Med, 27*(5), 1268-1272.

D'Anna-Hernandez, K. L., Ross, R. G., Natvig, C. L., & Laudenslager, M. L. (2011). Hair cortisol levels as a retrospective marker of hypothalamic–pituitary axis activity throughout pregnancy: comparison to salivary cortisol. *Physiology & behavior, 104*(2), 348-353.

Davenport, M. D., Tiefenbacher, S., Lutz, C. K., Novak, M. A., & Meyer, J. S. (2006). Analysis of endogenous cortisol concentrations in the hair of rhesus macaques. *General and comparative endocrinology, 147*(3), 255-261.

Kuehl, L. K., Hinkelmann, K., Muhtz, C., Dettenborn, L., Wingenfeld, K., Spitzer, C., . . . Otte, C. (2015). Hair cortisol and cortisol awakening response are associated with criteria of the metabolic syndrome in opposite directions. *Psychoneuroendocrinology, 51*, 365-370.

Manenschijn, L., Spijker, A. T., Koper, J. W., Jetten, A. M., Giltay, E. J., Haffmans, J., . . . van Rossum, E. F. (2012). Long-term cortisol in bipolar disorder: associations with age of onset and psychiatric co-morbidity. *Psychoneuroendocrinology, 37*(12), 1960-1968.

Mastromonaco, G. F., Gunn, K., McCurdy-Adams, H., Edwards, D., & Schulte-Hostedde, A. I. (2014). Validation and use of hair cortisol as a measure of chronic stress in eastern chipmunks (Tamias striatus). *Conservation Physiology, 2*(1), cou055.

Moya, D., He, M. L., Jin, L., Wang, Y., Penner, G. B., Schwartzkopf-Genswein, K. S., & McAllister, T. A. (2015). Effect of grain type and processing index on growth performance, carcass quality, feeding behavior, and stress response of feedlot steers. *Journal of animal science, 93*(6), 3091-3100.

Moya, D., Schwartzkopf-Genswein, K. S., & Veira, D. M. (2013). Standardization of a non-invasive methodology to measure cortisol in hair of beef cattle. *Livestock Science, 158*(1-3), 138-144.

Ouschan, C., Kuchar, A., & Mostl, E. (2013). Measurement of cortisol in dog hair: a noninvasive tool for the diagnosis of hypercortisolism. *Vet Dermatol, 24*(4), 428-431, e493-424.

Pulopulos, M. M., Hidalgo, V., Almela, M., Puig-Perez, S., Villada, C., & Salvador, A. (2014). Hair cortisol and cognitive performance in healthy older people. *Psychoneuroendocrinology, 44*, 100-111.

Sauve, B., Koren, G., Walsh, G., Tokmakejian, S., & Van Uum, S. H. M. (2007). Measurement of cortisol in human hair as a biomarker of systemic exposure. *Clinical and Investigative Medicine, 30*(5), E183-E191.

Schalinski, I., Elbert, T., Steudte-Schmiedgen, S., & Kirschbaum, C. (2015). The Cortisol Paradox of Trauma-Related Disorders: Lower Phasic Responses but Higher Tonic Levels of Cortisol Are Associated with Sexual Abuse in Childhood. *PloS one, 10*(8).

Steudte, S., Kirschbaum, C., Gao, W., Alexander, N., Schonfeld, S., Hoyer, J., & Stalder, T. (2013). Hair Cortisol as a Biomarker of Traumatization in Healthy Individuals and Posttraumatic Stress Disorder Patients. *Biological psychiatry, 74*(9), 639-646.

Steudte, S., Stalder, T., Dettenborn, L., Klumbies, E., Foley, P., Beesdo-Baum, K., & Kirschbaum, C. (2011). Decreased hair cortisol concentrations in generalised anxiety disorder. *Psychiatry research, 186*(2-3), 310-314.

Sumra, M. K., & Schillaci, M. A. (2015). Stress and the Multiple-Role Woman: Taking a Closer Look at the “Superwoman”. *PloS one, 10*(3), e0120952.

Tallo-Parra, O., Manteca, X., Sabes-Alsina, M., Carbajal, A., & Lopez-Bejar, M. (2015). Hair cortisol detection in dairy cattle by using EIA: protocol validation and correlation with faecal cortisol metabolites. *animal, 9*(06), 1059-1064.

Van Holland, B. J., Frings-Dresen, M. H. W., & Sluiter, J. K. (2012). Measuring short-term and long-term physiological stress effects by cortisol reactivity in saliva and hair. *International archives of occupational and environmental health, 85*(8), 849-852.

Vanaelst, B., Huybrechts, I., Bammann, K., Michels, N., De Vriendt, T., Vyncke, K., . . . De Henauw, S. (2012). Intercorrelations between serum, salivary, and hair cortisol and child-reported estimates of stress in elementary school girls. *Psychophysiology, 49*(8), 1072-1081.

Wippert, P.-M., Honold, J., Wang, V., & Kirschbaum, C. (2014). Assessment of chronic stress: comparison of hair biomarkers and allostatic load indices. *Psychology Research, 4*(7).

Xie, Q. Z., Gao, W., Li, J. F., Qiao, T., Jin, J., Deng, H. H., & Lu, Z. H. (2011). Correlation of cortisol in 1-cm hair segment with salivary cortisol in human: hair cortisol as an endogenous biomarker. *Clinical Chemistry and Laboratory Medicine, 49*(12), 2013-2019.

Yamanashi, Y., Morimura, N., Mori, Y., Hayashi, M., & Suzuki, J. (2013). Cortisol analysis of hair of captive chimpanzees (Pan troglodytes). *General and comparative endocrinology, 194*, 55-63.

Yu, T., Xu, H., Wang, W. W., Li, S. F., Chen, Z., & Deng, H. H. (2015). Determination of endogenous corticosterone in rodent's blood, brain and hair with LC-APCI-MS/MS. *Journal of Chromatography B-Analytical Technologies in the Biomedical and Life Sciences, 1002*, 267-276.
