# Supplementary material for: A systematic review of studies utilizing hair glucocorticoids as a measure of stress suggests the marker is more appropriate for quantifying short-term stressors
Source: Sci Rep. 2019 Aug 19;9:11997. doi: 10.1038/s41598-019-48517-2 (PMC6701156; doi:10.1038/s41598-019-48517-2)
Supplement: Supplementary file 3 — Supplemental Materials C (pre-registered protocol) [file 41598_2019_48517_MOESM3_ESM.pdf]

Supplemental Materials C. Pre-registered protocol

Supplement to:

A systematic review of studies utilizing hair glucocorticoids as a measure of stress suggests the marker is more appropriate for quantifying short-term stressors

Otto Kalliokoski[1], Finn K. Jellestad[2], Robert Murison[2]

[1] Department of Experimental Medicine, University of Copenhagen, Denmark

[2] Department of Biological and Medical Psychology, University of Bergen, Norway

SYSTEMATIC REVIEW PROTOCOL FOR ANIMAL INTERVENTION STUDIES

FORMAT BY SYRCLE (WWW.SYRCLE.NL)
VERSION 2.0 (DECEMBER 2014)

Item #	Section/Subsection/Item	Description	Check for approval
	A. General		
1.	Title of the review	Are we overestimating the utility of hair glucocorticoids? A systematic review exploring the empirical evidence supporting hair glucocorticoids as a measure of stress	
2.	Authors (names, affiliations, contributions)	• Finn K Jellestad; Department of Biological and Medical Psychology, University of Bergen; design of study, literature study, data extraction, quality assessment, data analysis<br>• Otto Kalliokoski; Department of Experimental Medicine, University of Copenhagen; design of study, literature study, data extraction, quality assessment, data analysis<br>• Robert Murison; Department of Biological and Medical Psychology, University of Bergen; design of study, literature study, data extraction, quality assessment, data analysis<br>• Kjersti Aksnes-Hopland; University Library, University of Bergen; development of search strategy	
3.	Other contributors (names, affiliations, contributions)	-	
4.	Contact person + e-mail address	Otto Kalliokoski, ohk@sund.ku.dk	
5.	Funding sources/sponsors	-	
6.	Conflicts of interest	None.	
7.	Date and location of protocol registration	Submitted: Jan. 12, 2016, Copenhagen, Denmark<br>Received/registered:	
8.	Registration number (if applicable)	-	
9.	Stage of review at time of registration	Immediately before literature searches.	
	B. Objectives		
	Background		
10.	What is already known about this disease/model/intervention? Why is it important to do this review?	Measuring glucocorticoids (GC) deposited in hair is rapidly becoming the new gold standard for biomarker-based stress measurements. With a quick adoption rate, in particular for studying humans, it has gone from being a curious alternative to being, seemingly, the preferred method in some fields. Gauging hypothalamic-pituitary-adrenal (HPA) axis functioning over time by measuring GC in other matrices such as blood, saliva, urine or fecal samples – the alternative, incumbent, methods – seems slated for obsolescence. It is easy to see why: hair is easily and painlessly sampled, it is an abundant source of material, a more hygienic matrix than the alternatives, and it has been argued to have superior qualities over other methods when it comes to gauging chronic stress.	

But can the technique live up to its promises? When using fecal glucocorticoids (FGC) as a proxy measure of stress one must consider factors such as how the GC are metabolized, the time scale from stressor to excretion of GC, how samples deteriorate over time, and how other factors, such as diet, affect the measurements; a daunting task which may lead to misinterpretations. With HGC we need to extend this list further to include the type, location and color of the hair, the confounding influences of sweating, washing and leaching; even meteorological factors such as ambient humidity have been shown to have an influence on the measured levels of GC in hair. Moreover, it has been established that the hair follicle has its own analogue of the HPA axis where extra-adrenal GC are produced locally in response to external stimuli. At present, there is no clear picture of how much of the GC found embedded in hair stem from local production versus how much is sequestered from the circulation. Perhaps even more concerning are the temporal aspects. With FGC, the time scale – how long after a stressful event the effects can be seen in fecal samples – can fairly easily be established using ACTH challenges or radiolabeled GC, whereas it is more complicated for hair. Hair growth is a fairly constant process, and it has been suggested that GC are deposited at the root of the hair – the hair becoming a historical record of preceding levels of HPA axis functioning (often interpreted in a simplified framework as physiological stress). It then follows that life events can be studied by following the individual strand of hair – from events of last week, close to the root of the hair, to events months in the past, represented by the tip of the hair (the longer the hair, the longer this "stress calendar" stretches into the past). Whereas this reasoning has been used when studying individuals over time, no one has, in a controlled environment, been able to prove this hypothesis. In fact, multiple studies have shown that the GC levels measured in hair are more reflective of stressful events in the recent past – hours, or even minutes, in the past.

In addition, as hair samples are effortlessly obtained, one sample can, through longitudinal sectioning, be subdivided into many for a generous data material and as GC content can be analyzed quickly with a minimum of sample pre-processing, hypotheses can be tested at comparatively blinding speeds. With some animal studies seeming to have been conceived and carried out in a matter of days (hair sampling is non-invasive and does in most cases not require legal or ethical approval and can be tacked onto nearly any pre-existent experiment) there is a considerable risk of an accumulating publication bias if only positive findings make their way into scientific

		journals.<br><br>Despite the many uncertainties surrounding the method, it is presently used to gauge mental illness, the wellbeing of human trauma victims, PTSD sufferers, and children; to assess animal welfare in wildlife, captive animals and laboratory animals. The mismatch between the uncertainties of the method and the confidence with which it is applied is concerning. Although it is worth emphasizing that most researchers will err on the side of caution in their studies – not risking the wellbeing of people or animals, even when elevated levels of GC are not found – if we do not explore the limits of the method and critically assess the findings within the field, the poorly conducted studies will undermine the solid findings; the field of biomarkers for studying stress risks going the way of the Boy Who Cried Wolf.	
	Research question		
11.	Specify the disease/health problem of interest	Elevated HPA axis functioning, suggested to be related to stress and/or stress disorders.	
12.	Specify the population/species studied	All vertebrates including humans	
13.	Specify the intervention/exposure	Stress – physiological or psychological.	
14.	Specify the control population	No stress/lower levels of stress	
15.	Specify the outcome measures	GCs measured in hair	
16.	State your research question (based on items 11-15)	Does empirical evidence support the current use of hair glucocorticoids for gauging stress?	
	C. Methods		
	Search and study identification		
17.	Identify literature databases to search (*e.g.* Pubmed, Embase, Web of science)	Databases: Medline, Web of Science, EMBASE, Zoological Record, PsycINFO	
18.	Define electronic search strategies (*e.g.* use the step by step search guide[15] and animal search filters[20, 21])	Search terms have been attached as an appendix to the protocol (Appendix 1).	
19.	Identify other sources for study identification	☐Reference lists of included studies ☐Books<br>☐Reference lists of relevant reviews<br>☐Conference proceedings, namely:<br>☐Contacting authors/organisations, namely:<br>☒Other, namely: Citing articles.	
20.	Define search strategy for these other sources	Citing articles: Following title/abstract screening, the utilized databases are used to identify papers that reference the determined-to-be-relevant papers. Any paper identified that has not been previously rejected is included in the next step.	
	Study selection		

21.	Define screening phases (*e.g.* pre-screening based on title/abstract, full text screening, both)	Screening steps:<br>1) Pre-screening based on title/abstract.<br>2) Full text screening.	
22.	Specify (a) the number of reviewers per screening phase and (b) how discrepancies will be resolved	(a) Pre-screening: Three reviewers<br>Full text screening: Three reviewers<br>(b) Pre-screening: If there is a disagreement over the relevance of a paper, it is kept for the full text screening.<br>Full text screening: If there is a disagreement over the relevance of a paper, a consensus decision is made in a meeting of the three reviewers.	
	Define all inclusion and exclusion criteria based on:		
23.	Type of study (design)	Inclusion criteria: Control/experimental design ("case-control"), Hair GC correlated to GCs in another matrix.<br>Exclusion criteria: Review papers, opinion papers, no control group/condition present. Although closely related matrices such as nails, talons, baleen, etc. have been used in some studies, we will focus this review on hair exclusively and exclude other keratinous matrices at current.	
24.	Type of animals/population (*e.g.* age, gender, disease model)	Inclusion criteria: Vertebrates (w/ hair)<br>Exclusion criteria: Non-vertebrates, vertebrates without hair.	
25.	Type of intervention (*e.g.* dosage, timing, frequency)	Inclusion criteria: Stress, natural or induced (Acute/intermittent/chronic/PTSD)<br>Exclusion criteria: Pharmacological studies, excepting ACTH or CRH manipulation.	
26.	Outcome measures	Inclusion criteria: Hair GC<br>Exclusion criteria: Not hair GC	
27.	Language restrictions	Inclusion criteria: English language papers<br>Exclusion criteria: Papers that cannot confidently be translated into English.	
28.	Publication date restrictions	Inclusion criteria: -<br>Exclusion criteria: Papers published after review initiation. Papers published online ahead of journal publication will however be used if they are indexed in the utilized journal databases.	
29.	Other	Inclusion criteria: Peer-reviewed papers<br>Exclusion criteria: Full text not available, inadequate documentation	
30.	Sort and prioritize your exclusion criteria per selection phase	Title/abstract screening: Steps 24, 26 and 29<br>Full text screening: Steps 23, 25, 27, 28 and 29	
	Study characteristics to be extracted (for assessment of external validity, reporting quality)		
31.	Study ID (*e.g.* authors, year)	Authors, year, journal.	
32.	Study design characteristics (*e.g.* experimental groups, number of animals)	Type of study (Experimental or Correlational), experimental groups, number of animals in each group.<br>We define the types of study as follows:<br>Exploratory – the relation of hGC to one or multiple factors is explored. There is no clear a priori hypothesis to subdivide study population(s) into stress and control populations.	

		Experimental – The relation of hGC to one or multiple factors is explored. There is a clear a priori hypothesis and the study population has been subdivided into individuals of (purportedly) higher and lower levels of stress.<br>Correlational – hGC is correlated to GC in another biological matrix (blood, saliva, urine, feces) in the same individuals.<br>Other – study design does not fulfil the criteria of any of the above.<br><br>Only experimental and correlational studies are included for assessment of external validity and reporting quality.	
33.	Animal model characteristics (*e.g.* species, gender, disease induction)	Species (listed as fully as possible), gender, age	
34.	Intervention characteristics (*e.g.* intervention, timing, duration)	Type of stressor, duration (acute/intermittent/chronic), latency to measurement,	
35.	Outcome measures	Cortisol or corticosterone	
36.	Other (*e.g.* drop-outs)	Full list of extracted characteristics has been attached as an appendix (Appendix 2) to the protocol.	
	Assessment risk of bias (internal validity) or study quality		
37.	Specify (a) the number of reviewers assessing the risk of bias/study quality in each study and (b) how discrepancies will be resolved	a) 3 reviewers.<br>b) Discrepancies will be settled in a consensus meeting.	
38.	Define criteria to assess (a) the internal validity of included studies (*e.g.* selection, performance, detection and attrition bias) and/or (b) other study quality measures (*e.g.* reporting quality, power)	☐By use of SYRCLE's Risk of Bias tool[4]<br>☐By use of SYRCLE’s Risk of Bias tool, adapted as follows:<br>☐By use of CAMARADES' study quality checklist, e.g [22]<br>☐By use of CAMARADES' study quality checklist, adapted as follows:<br>☒Other criteria, namely: A checklist adapted specifically for the study has been developed and attached as an appendix (Appendix 3) to the protocol.	
	Collection of outcome data		
39.	For each outcome measure, define the type of data to be extracted (*e.g.* continuous/dichotomous, unit of measurement)	For experimental studies:<br>Continuous data are extracted for both stress and control groups.<br>Groups. In order to consider all the empirical evidence, the control group can consist of the same animals as the stress group, just under different circumstances. Repeated measures-designs are however problematic in a meta-analysis context; we will therefore only resort to this when a true control group is not included in the study.<br>Multiple measurements. If multiple sampling sites (for example right leg and left leg) are included in the design, we will construct a weighted average (weighted by the number of samples or, if this cannot be determined, by the number of sampling sites assuming equal distribution between sites) of the comparisons (stress to control) to not inflate the weight of any one study.<br>Units. hGC values in moles or grams per weight of hair	

		(e.g. nmol/mg or mg/g) are extracted. We will also accept non-standardized raw data from e.g. antibody-based assays; i.e. measures expressed as absorbance (as these can be linearly translated to the quantities above, making the inter-group distances directly comparable).<br>Central tendency. Acceptable measures are mean (arithmetic or geometric) or median. If multiple measures are present, geometric mean (mean on a logarithmic axis) or median are preferred due to GC often tending toward a log-normal distribution.<br>Measure of dispersion. Acceptable measures are standard deviation, standard error of the mean (if population size is listed), 95% (or similar) confidence intervals, or interquartile range. If data is extracted from figures and whiskers are not defined, we will assume that the measure is SD, as the more conservative measure (compared to SEM) is less likely to bias the analysis.<br>Transformation into standardized mean difference:<br>We must make the assumption that the authors of each publication have chosen their measures of central tendency and dispersion appropriately. We thus assume that means have been used for data conforming to a normal distribution, whereas medians or geometric means have been used for log-normal distributions (where median = geometric mean). Since the meta-analyses assume symmetrical distributions around a mean, log-normal data will be transformed. Data is summarized as means with standard deviations before standardized mean differences are computed.<br><br>For correlational studies:<br>Statistical test data is extracted from correlational studies, rather than raw data. Correlation coefficients and the number of data points are extracted. A measure of dispersion for the correlation coefficient is also extracted if present (standard deviation or 95% CI), or data from the statistical significance testing is used to approximate one.	
40.	Methods for data extraction/retrieval (*e.g.* first extraction from graphs using a digital screen ruler, then contacting authors)	Data are extracted from text/tables. If raw data cannot be found in text form, results are extracted from graphs using a digital screen ruler. If this is ambiguous or gives too low-precision results, the corresponding author of the paper is contacted by email. If no answer can be obtained within two weeks, the paper may be excluded from the study.	
41.	Specify (a) the number of reviewers extracting data and (b) how discrepancies will be resolved	a) 3 reviewers<br>b) Discrepancies will be settled in a consensus meeting.	
	Data analysis/synthesis		
42.	Specify (per outcome measure) how you are planning to combine/compare	Descriptive data will be used to describe the state of the field. How many studies, yearly, utilize hGC as a measure	

	the data (*e.g.* descriptive summary, meta-analysis)	of stress? How many experimental studies have been published in relation to e.g. exploratory studies (i.e. is the use of hGC well-founded)? Which methods and study designs are most prevalent, etc.<br>Meta-analysis will be used to evaluate the empirical evidence supporting the use of hGC as a measure of stress. We will do this by studying hGC in stressed individuals relative to controls, but also by evaluating the evidence that hGC correlates to GC in other matrices (that have previously been validated as measures of stress). Since it may be that hGC is applicable in one context, but not another, we will also stratify data by the type of stressor to evaluate when and how hGC are applicable.	
43.	Specify (per outcome measure) how it will be decided whether a meta-analysis will be performed	Experimental studies: Meta-analysis of hGC from stress studies will be carried out only if data can be extracted from at least ten studies (discounting PTSD studies, since this effect may go the opposite direction) or five studies of the same type (acute/intermittent/chronic/PTSD).<br>Correlations: Meta-analysis of correlation coefficients will only be carried out for those correlations for which data can be extracted from at least four studies.	
	If a meta-analysis seems feasible/sensible, specify (for each outcome measure):		
44.	The effect measure to be used (*e.g.* mean difference, standardized mean difference, risk ratio, odds ratio)	We will employ standardized mean differences for hGC data to ensure that all measurements are brought onto a comparable scale.	
45.	The statistical model of analysis (*e.g.* random or fixed effects model)	We will use a random effects model as we expect the stressors to be of different magnitude (i.e. the different study designs will not be directly comparable).	
46.	The statistical methods to assess heterogeneity (*e.g.* I^2, Q)	Study heterogeneity will be assessed by calculating the I^2 statistic.	
47.	Which study characteristics will be examined as potential source of heterogeneity (subgroup analysis)	The meta-analysis of experimental studies will be stratified by (1) type of stressor (Acute/intermittent/chronic/PTSD), (2) by human/non-human and, if the number of studies allows it, (3) by study quality assessment parameters (to evaluate whether there are obvious methodological characteristics or risks of bias that heavily contribute to study heterogeneity).<br>The meta-analysis of correlational studies will be attempted only through subgroup analysis, stratified by biological matrix.	
48.	Any sensitivity analyses you propose to perform	Subgroup analysis will be undertaken to test the robustness of the meta-analysis. If the number of studies is sparse and does not lend itself to subgroup analysis, leave-one-out analysis will be used to gauge robustness.	
49.	Other details meta-analysis (*e.g.* correction for multiple testing, correction for multiple use of control group)	In the cases where multiple sampling sites on subjects have been employed (e.g. sampling from chest, back, arms etc.) in experimental studies we will calculate an average standardized mean difference between the experimental and control groups (weighted by sample size).	
50.	The method for assessment of publication bias	Funnel plot (with trim and fill, if enough data can be obtained to motivate its use) and Egger regression.	

Final approval by (names, affiliations):	Otto Kalliokoski, Department of Experimental Medicine, University of Copenhagen (on behalf of the four authors)	Date: Jan 12 2016

Appendix 1. Search strategy details

The following databases and search terms are used to identify potentially relevant studies. Subject heading searches – e.g. MeSH terms – did not produce additional "hits" and were thus excluded for cleaner and more transparent search terms. The most up-to-date version of each database was searched. The searches were all carried out on January 13, 2016.

1. Embase/Medline/PsycINFO

Access (through Ovid):

gateway.ovid.com/autologin.cgi

Search field definition:

((hair OR fur OR coat OR "hair follicle" OR whisker*) AND (cortisol OR corticostero* OR glucocortico* OR adrenocortic*) AND (stress* OR allosta* OR PTSD OR anxiety OR depress*))*

Type of search:

MP Mapping Alias

2. Web of Science (Core Collection)/Zoological Record

Access (through Web of Science):

Web of Science:
http://apps.webofknowledge.com/WOS_GeneralSearch_input.do?product=WOS&SID=Q2Gz7ZGQf27LYjBZ47F&search_mode=GeneralSearch

Zoological Record:
http://apps.webofknowledge.com/ZOOREC_GeneralSearch_input.do?product=ZOOREC&search_mode=GeneralSearch&SID=Y2mQTWG2cIxCZZrDAOJ&preferencesSaved=

Search term:

((“hair” OR “fur” OR “coat” OR "hair follicle" OR whisker*) AND (“cortisol” OR corticostero* OR glucocortico* OR adrenocortic*) AND (stress* OR allosta* OR “PTSD” OR “anxiety” OR depress*))*

Search field definition:

Topic

Appendix 2. Assessment of external validity and reporting quality

Study characteristics		Comments
1. List study ID	Authors, year, journal	
2. What type of study is this?	Experimental/correlational	
3. What are the subjects?	Human (infants/adults/etc.), non-human animals (species?)	
4. What type of stressor is studied?	Acute stressor/intermittent stressor/chronic stressor/PTSD/Not applicable (correlational studies only)<br>Describe stressor in comments.	
**Subject selection**		**Comments**
5. How many subjects were included in the study?	List cohorts in comments	
6. Were the group sizes determined by an a priori power analysis according to the report?	Yes/no	
**Subject information**		**Comments**
7. What sex were the subjects?	Male/female/mixed/unclear	
8. What other subject characteristics are listed?	Age? Health conditions? Other characteristics?	
9. For heterogeneous cohorts, do the researchers account for the heterogeneity in their tests?	Yes/no/unclear/not applicable (e.g. homogeneous groups)	
10. Were relevant medications listed for the subjects?	Yes/no/unclear/not applicable	
**Sampling**		**Comments**
11. Were multiple sites on the body sampled?	Yes (list number of sites in comments)/no/unclear	
12. How were hairs sampled?	Whole hair with follicle/whole hair without follicle/segment (lock) of hair/unclear	
13. Were the hairs further segmented to create sub-samples?	Yes/no/unclear	
**Sample processing**		**Comments**
14. Were the hairs washed prior to extraction of GCs?	Yes (list washing medium in comments)/no/unclear	
15. How were the hairs processed prior to extraction?	Cut/milled or mortared/no processing/other (list in comments)	
16. What extraction medium was used?	Methanol/ethanol/propanol/other (list if medium is diluted, e.g. 50% methanol)	
**Analysis**		**Comments**

17. What GCs were measured?	Cortisol/corticosterone/other (e.g. relevant metabolites)	
18. What analysis method was utilized?	Antibody based (ELISA/EIA/RIA – list kit/antibody when possible)/chromatography (GC/LC/HPLC)/mass spectrometry (MS/HPLC-MS)/other	

Appendix 3. Risk of bias checklist (assessment of internal validity)

Adapted from:

1. Hartling L, Bond K, Harvey K, et al. Developing and testing a tool for the classification of study designs in systematic reviews of interventions and exposures. Prepared by the University of Alberta Evidence-based Practice Center under Contract No. 290-02-0023. Rockville, MD: Agency for Healthcare Research and Quality: June 2009. AHRQ Publication No. 11-EHC007-EF.
2. Viswanathan M, Ansari MT, Berkman ND, Chang S, Hartling L, McPheeters LM, Santaguida PL, Shamliyan T, Singh K, Tsertsvadze A, Treadwell JR. Assessing the Risk of Bias of Individual Studies in Systematic Reviews of Health Care Interventions. Agency for Healthcare Research and Quality Methods Guide for Comparative Effectiveness Reviews. March 2012. AHRQ Publication No. 12-EHC047-EF. Available at: www.effectivehealthcare.ahrq.gov/

**Selection bias**		**Comments**
1. Were cases and controls selected appropriately?	Yes/No/Unclear	
2. Does the design or analysis control account for important confounding and modifying variables through matching, stratification, multivariable analysis, or other approaches?	Yes/No/Unclear	
**Performance bias**		**Comments**
3. Did researchers rule out any impact from a concurrent intervention or an unintended exposure that might bias results?	Yes/No/Unclear	
**Attrition bias**		**Comments**
4. Were missing data handled appropriately?	Yes/No/Unclear	
**Detection bias**		**Comments**
5. Were the cases and controls assessed concurrently under similar ambient conditions?	Yes/No/Unclear	
6. Were the outcome assessors blinded?	Yes/No/Unclear	
7. Were stressors assessed/defined using valid and reliable measures, implemented consistently across all study participants?	Yes/No/Unclear	
8. Were confounding variables assessed using valid and reliable measures, implemented consistently across all study participants?	Yes/No/Unclear	
**Reporting bias**		**Comments**
9. Are the results of all analyses reported (or data made available)?	Yes/No/Unclear	
**Summary:**	Yes:<br>No:<br>Unclear:	

Appendix 4. Change log

For full transparency we have listed the changes made to the original protocol below.

January 12, 2016
Original protocol submitted. Initial search of databases is performed.

January 13, 2016
Actual search is performed (as an error was discovered in the Web of Science database selection for the search performed on the 12th of January). The link to Web of Science was also updated to amend this.
